# Complex, low‐intensity, individualised naturalistic developmental behavioural intervention in toddlers and pre‐schoolers with autism spectrum disorder: The multicentre, observer‐blind, parallel‐group randomised‐controlled A‐FFIP trial

**DOI:** 10.1111/jcpp.14162

**Published:** 2025-03-26

**Authors:** Christine M. Freitag, Marietta Kirchner, Lukas D. Sauer, Solvejg K. Kleber, Leonie Polzer, Naisan Raji, Christian Lemler, Ulrike Fröhlich, Tomasz Jarczok, Julia Geissler, Franziska Radtke, Melanie Ring, Veit Roessner, Regina Taurines, Michelle Noterdaeme, Karoline Teufel, Ziyon Kim, Janina Kitzerow‐Cleven

**Affiliations:** ^1^ Department of Child and Adolescent Psychiatry, Psychosomatics and Psychotherapy Autism Research and Intervention Centre of Excellence, University Hospital Frankfurt, Goethe‐University Frankfurt am Main Germany; ^2^ Institute of Medical Biometry, Heidelberg University Hospital Heidelberg Germany; ^3^ Department of Child and Adolescent Psychiatry, Psychosomatics and Psychotherapy Josefinum Augsburg Germany; ^4^ Department of Child and Adolescent Psychiatry, Psychosomatics and Psychotherapy Centre for Mental Health, University Hospital of Wuerzburg Wuerzburg Germany; ^5^ Department of Child and Adolescent Psychiatry Technische Universität Dresden Dresden Germany

**Keywords:** Naturalistic, developmental, behavioural, autism, social communication, repetitive behaviour, randomised‐controlled

## Abstract

**Background:**

Naturalistic developmental behavioural interventions (NDBI) may improve social communication in toddlers/pre‐school aged children with autism spectrum disorder (ASD). Here, we study efficacy of the low‐intensity, complex NDBI ‘Frankfurt Early Intervention Program for ASD’ (A‐FFIP) over 1 year by a confirmatory phase‐III, prospective, randomised, controlled, parallel‐group study with two treatment arms over four centres.

**Methods:**

*Main inclusion criteria*: ASD (DSM‐5), age 24–66 months, developmental quotient >30. *Intervention*: Manualised A‐FFIP intervention. *Control intervention:* Early intervention as usual (EIAU). *Primary outcome*: Change in core ASD symptoms from baseline (T2) to immediate intervention endpoint at 12 months (T6) based on the blindly rated Brief Observation for Communication Change (BOSCC) total score. *Statistical analysis:* Mixed model for repeated measures with covariates baseline BOSCC‐total, chronological age and centre.

**Results:**

Between July 2018 and October 2021, *N* = 134 children with ASD were randomly allocated to intervention (A‐FFIP: *n* = 68, EIAU: *n* = 66). Groups did not differ at baseline, with a mean age of 49 (*SD* 10) months, a mean developmental age of 23.3 (*SD* 13.6) months and 26 (19.4%) females. The SARS‐CoV‐2 pandemic interfered severely with trial procedures. Intention‐to‐treat analysis in the primary analysis set, with at least one postbaseline BOSCC measure (A‐FFIP *n* = 64, EIAU *n* = 60), did not find differences in the primary outcome by group (adjusted ES −0.06, 95% CI to −0.24 to 0.11). SARS‐CoV2‐related lockdown led to less improvement across groups. Secondary outcomes showed stronger improvements in parent‐rated repetitive behaviour as well as parent‐ and teacher‐rated executive functions for A‐FFIP versus EIAU. Adverse events were comparable between groups.

**Conclusions:**

The manualised NDBI program A‐FFIP, which allows individually targeting six core basic abilities and five developmental domains related to longitudinal development in ASD, did not improve social communication, cognitive or behavioural outcomes beyond EIAU after 1 year, but may improve repetitive behaviour and executive function.

## Introduction

Autism spectrum disorder (ASD) is a neurodevelopmental disorder characterised by impaired social communication and stereotyped, repetitive behaviour. Toddlers and pre‐schoolers show a variable development of core symptoms, cognitive and language abilities, and additional behavioural. Naturalistic developmental behavioural interventions (NDBI) are likely to improve social communication (Sandbank et al., 2023). They are based on developmental and behavioural theories and work with natural antecedents and rewards within naturally occurring situations. Complex programs assume multiple components or mechanisms of change, and target several developmental areas, such as social communication, cognition, language and adaptive behaviour (Skivington et al., 2021). Focussed programs target just one mechanism, such as joint attention. In addition, NDBI vary in intervention intensity. For example, the therapist‐implemented Early Start Denver Model (ESDM) is a high intensity (>20 hr/week) (Dawson et al., 2010), while the parent‐implemented pivotal response treatment (PRT) a low‐intensity (<5 hr/week) complex NDBI (Uljarević et al., 2021). Examples of low‐intensity, focussed programs are JASPER (Kasari et al., 2010) and PACT (Green et al., 2010).

In Europe, mainly low‐intensity interventions are publicly funded for young children with ASD (Salomone et al., 2016). Thus, it is possible to study low‐intensity approaches by randomised‐controlled trials (RCT) with comparable active control conditions. Based on the locally available funding of maximum 2 hr of ASD‐specific early intervention per week, we have developed the ‘Frankfurt Early Intervention Program for ASD’ (A‐FFIP), which is a low‐intensity, complex NDBI targeting several developmental areas in addition to social communication. Within an individually applied naturalistic developmental behavioural, but still highly structured and manualised approach (Teufel et al., 2017), A‐FFIP allows to flexibly address the child's individual level of abilities. It targets six core basic abilities, such as joint attention, imitation or planning, and five developmental domains, such as communication, emotion regulation or adaptive behaviour, which have been shown to be affected in young children with ASD and may influence longitudinal outcome (Bottema‐Beutel, 2016; Green & Carter, 2014; Hendrix et al., 2022; Iao et al., 2024; Yeung et al., 2024). A‐FFIP allows addressing developmental variability and heterogeneity within ASD by choosing exercises targeting the individual child's current basic abilities and developmental domain level. Additional behavioural issues are flexibly addressed by evidence‐based behavioural interventions. Parents are involved in the intervention sessions and are encouraged to practise regularly at home. In an observer‐blind, non‐randomised, small case–control study, a medium effect on core ASD symptoms was found after 1 year of A‐FFIP intervention compared with early intervention as usual (Kitzerow, Teufel, et al., 2020).

Here, we report results of the much larger multicentre, well‐powered, single‐blind RCT of A‐FFIP over 1 year and its effects on ASD core symptoms as the primary outcome measured by the blindly rated BOSCC (Grzadzinski et al., 2016; Kitzerow et al., 2016). Secondary outcomes cover a broad range of the child's competencies and family‐related aspects. During the study, the SARS‐CoV2 pandemic occurred, coming along with severe disruptions to the children's and families’ lives, treatment interruptions as well as other obligations affecting treatment. We thus also aim at addressing pandemic‐induced effects by sensitivity analyses. Characteristics of children and families were examined as potential moderators in an exploratory fashion. The study has been preregistered with the German Clinical Trials Register (Deutsches Register Klinischer Studien, DRKS; ID: 00016330), and the study protocol has been published (Kitzerow, Hackbusch, et al., 2020).

## Methods

This confirmatory phase‐III, prospective, randomised, multicentre, controlled, parallel‐group study with two treatment arms, an allocation ratio of 1:1 and six measurement time points was approved by the ethical committee of the medical faculty of Goethe University at Frankfurt and successively by the local ethical committees of the other study centres at Augsburg, Dresden and Würzburg (for more details, see S4 in Appendix S1).

### Inclusion criteria

Diagnosis of autism spectrum disorder according to DSM‐5, confirmed by the Autism Diagnostic Interview (ADI‐R; <4 years old: toddler algorithm) (Bölte et al., 2008; Kim et al., 2013) and the Autism Diagnostic Observation Schedule‐2 (ADOS‐2) (Lord, 2015); age 24–66 months old; parents willing and able to participate regularly in the intervention; written informed consent of the child's legal guardians.

### Exclusion criteria

Non‐verbal developmental quotient (DQ) or intelligence quotient (IQ) ≤30, non‐verbal mental age ≤12 months; diagnosed vision or hearing impairments interfering with intervention, cerebral palsy, chronic neurological/neurodegenerative disorder, unstable epilepsy, Rett's or Angelman's syndrome, (history of) severe psychosocial deprivation, insufficient care by parents, attachment disorder, institutional upbringing, parents not verbally fluent in German and/or unable to read German.

### A‐FFIP intervention

A‐FFIP is a manualised, published intervention (Teufel et al., 2017). The manual provides detailed information for therapists on the recommended intensity (twice 1 hr of intervention/week), standardised setting, work with parents, how to establish personalised intervention targets for the child and related intervention methods. For details, see S2–S3 in Appendix S1.

Prior to starting the A‐FFIP intervention at the four centres, therapists were trained extensively in the manualised application of A‐FFIP. Intervention sessions of children not included in the study were rated from video by the A‐FFIP‐specific intervention adherence coding scheme. Prespecified criteria, that is, at least 50 of a maximum of 56, had to be met by all therapists before providing A‐FFIP within the trial. During the intervention, continuous training and standardised supervision were provided, based again on videos of single sessions, which were obtained every 7th to 10th intervention session and blindly rated by the A‐FFIP‐specific intervention adherence coding scheme (Kitzerow, Hackbusch, et al., 2020). The number of therapists at the different centres was as follows: Frankfurt 12 (20 co‐therapists), Augsburg 6 (5 co‐therapists), Dresden 4 (2 co‐therapists), Wuerzburg 5 (7 co‐therapists). An average of 16 (*SD* 11, range 3–42) videos of intervention sessions per child were coded for manual adherence, and individual feedback was provided to the therapists. The therapists’ manual adherence was over 95% (mean 1.92, *SD* 0.05; complete manual adherence = 2; broken down by centre: Appendix S2: Table S3).

### Active control intervention

Early intervention as usual (EIAU) comprises individual or group‐based ASD‐specific or unspecific early intervention with an intensity of ≤10 hr/week.

For both, A‐FFIP and EIAU, additional speech and language, occupational or other non‐ASD‐specific therapeutic interventions, individual support at kindergarten and stable psychotropic medication were allowed and continuously documented during the study period.

### Outcome measures

Outcomes were obtained prior to randomisation (T1 enrolment/T2 baseline), after 3 months (T3; only questionnaires), after 6 months (T4, all assessments), after 9 months (T5, only questionnaires) and after 12 months (T6, all assessments). Due to the SARS‐CoV‐2 pandemic, T1/T2 to T6 measurements could not be obtained exactly at the prespecified time points, with a minimum of 26 and a maximum of 51 weeks (median 28, Q1–Q3 27–34) between T2 and T4, and a minimum of 52 and a maximum of 77 weeks (median 59, Q1–Q3 55–63) between T2 and T6 measurements.

The prespecified *primary outcome* is the change in the Brief Observation for Communication Change (BOSCC) – total score between T6 and T2, based on the research version of 11 Dec 2017 (Grzadzinski et al., 2016; Kitzerow et al., 2016), which was blindly obtained at T2, T4 and T6. The BOSCC captures ASD‐specific symptoms based on a semi‐standardised play session of the child with an adult play partner. Here, we chose the most distant setting to the intervention, namely a free play interaction with an unfamiliar, blinded tester. The BOSCC was blindly rated from standardised videos by independent raters based in Frankfurt. Pairwise inter‐rater reliability (intraclass correlation efficient = ICC) for the BOSCC‐total was 0.955 (95% CI 0.953 to 0.957), based on 70 patients and 140 ratings.

Prespecified child, parent and family‐related *secondary outcomes* were also obtained (details see S5 in Appendix S1). *Child‐related measures* comprised the BOSCC social communication (SC) and repetitive behaviour (RRB) subdomains, the blindly obtained and blindly rated ADOS‐2 comparison (i.e. calibrated severity score, CSS), social affect (SA) and repetitive behaviour scores (RRB) (Lord, 2015), the SRS‐16 item version (Sturm et al., 2017) and the RBS‐R (Kästel et al., 2021) filled in by parents and teachers. Cognitive skills were assessed by blinded testers via the Bayley Scales of Infant and Toddler Development – Third Edition (Bayley‐ III) (Reuner, 2015) or Wechsler Preschool and Primary Scale of Intelligence (WPPSI‐III) (Petermann, 2014). The Child Behaviour Checklist 1½‐5 (CBCL1½‐5) and the Teacher Report Form 1½‐5 (C‐TRF) (Plück et al., 2022) were filled in by parents (CBCL1½‐5) or kindergarten teachers (C‐TRF) to assess internalising and externalising psychopathology. Executive functioning was measured by the parent‐ and teacher‐rated BRIEF‐P (Daseking & Petermann, 2013). *Parent‐ and family‐related measures* comprised the Parenting Sense of Competence Scale (PSOC) (Kliem et al., 2014) and the Depression Anxiety and Stress Scales– short form (DASS‐21) (Nilges & Essau, 2015). Quality of life was rated by an adapted version of the Family quality of Life Survey‐2006 ID/DD version (FQOLS‐2006‐ID/DD) via a semi‐standardised interview (Perry & Isaacs, 2015).

### Information on medical conditions, intervention, education and family

By a semi‐standardised parent interview, information on the child's medical conditions, psychotropic and other medication, and additional psychosocial interventions was obtained at each assessment (T1/T2 to T6). Psychosocial support at kindergarten or school and early intervention available in Germany were coded with respect to the nature of support, weekly intensity and start/ending date. The child's sex, number of siblings, parental education status (ISCED‐2011, UNESCO), languages spoken at home and family migration status (i.e. at least one parent had immigrated as adult to Germany) were documented.

### Adverse events

Adverse events were enquired about at least every 3 months by a semi‐standardised parent interview or coded when they were reported by parents during any of the intervention sessions (A‐FFIP) or via parent information (EIAU) between assessment time points.

### Randomisation

Patients were randomised in a 1:1 ratio via a centralised web‐based tool (randomizer.at), set up by the data management. Block randomisation with variable block lengths stratified by centre and sex were performed via the web‐based tool by the enrolling clinicians in the respective centres.

### Sample size

In a 1‐year non‐randomised‐controlled trial (Kitzerow, Teufel, et al., 2020), an effect size of 0.61 of A‐FFIP versus EIAU on core ASD symptom improvement assessed via the ADOS‐CSS (Kitzerow, Teufel, et al., 2020), and a drop‐out rate of 20% were observed. For the present study, a slightly more conservative effect size of 0.55 was chosen for the primary outcome BOSCC‐total, similarly capturing core ASD symptoms (Kitzerow et al., 2016). With alpha = 5% (two‐sided) and 1‐beta = 80%, a sample size of 106 (2×53) was required to detect an effect size of 0.55 with the two‐sample *t*‐test (ADDPLAN, version 6.1.1). To compensate for the potential loss of information caused by a 20% drop‐out/loss‐to‐follow‐up, the total number of randomised children was *N* = 134 (2×67).

### Statistical methods

A statistical analysis plan (SAP) was finalised prior to database closure and data analysis (provided by request). Descriptive data of baseline‐, intervention‐ and outcome‐related variables are reported for the intention‐to‐treat population (ITT), based on the full analysis set (FAS), and comprise mean, standard deviation, median, quartiles and range for continuous variables, and absolute and relative frequencies for categorical variables. The primary analysis was performed in the ITT population for whom at least one of the T4 or T6 BOSCC‐total scores was measured (primary analysis set [PAS]). The analysis consisted of fitting a mixed model for repeated measures (MMRM) of the BOSCC‐total at T4 and T6 adjusted for the covariates baseline BOSCC‐total, chronological age and centre, with an interaction term of treatment group and time of BOSCC measurement in weeks, taking patient‐specific random effects with power spatial covariance structure into account. The confirmatory analysis comprised testing the adjusted group difference of the MMRM least square means at 52 weeks after baseline (two‐sided type I error rate 5%). An adjusted effect size measure was derived from the t‐statistic of the confirmatory test. The effect size's confidence interval was obtained via bootstrap and may hence not be fully coherent with the *p*‐value. Secondary endpoints were studied similarly. If secondary endpoints were measured at more than two postbaseline visits, then all available measurements were included in the model. Several effect sizes with their 95% confidence intervals were reversed (multiplication by −1), namely the BOSCC, ADOS, RBS‐R, CBCL, BRIEF‐P and DASS‐21 scales, to achieve homogeneity with respect to the interpretation, that is, positive effect sizes are related to improvement by A‐FFIP. The moderator analysis also employed MMRM and additionally included three‐fold interaction terms of time, treatment group and the respective moderator. Concerning safety, rates of (serious) adverse events were calculated in the full randomised sample (full analysis set). For the rate of children with an adverse event, 95% Wilson confidence intervals were reported. The software package SAS® Version 9.4 (SAS Inc., Cary/NC, USA) was used for statistical analyses. Most figures were generated on a validated R server using R version 4.2.2 (2022‐10‐31). For further details, see S6 in Appendix S1.

## Results

**Figure 1:**
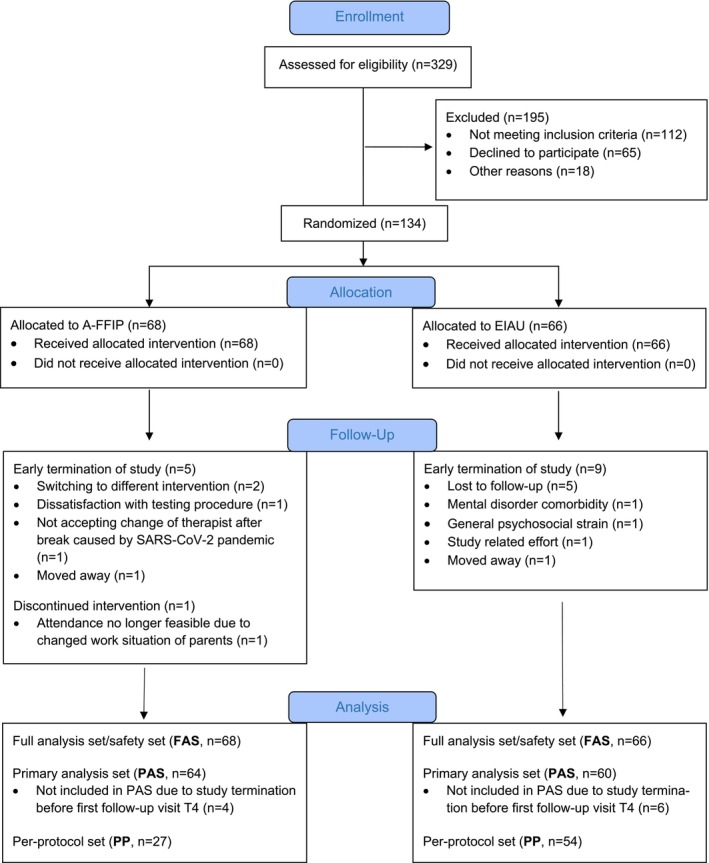
A‐FFIP: CONSORT flow diagram

Between 3 July 2018 and 6 October 2021, the planned number of *N* = 134 children with ASD was randomised. *N* = 329 children were screened (Frankfurt *n* = 117, randomised *n* = 72 (62%), Augsburg *n* = 54, randomised *n* = 28 (52%), Dresden *n* = 121, randomised *n* = 13 (11%), Wuerzburg *n* = 37, randomised *n* = 21 (57%)); *n* = 112 did not meet inclusion criteria, *n* = 65 declined to participate and *n* = 18 were not randomised due to other reasons. For information on the criteria for the full analysis set (FAS), the primary analysis set (PAS) and the per‐protocol set (PP), see Figure 1, CONSORT Flow Diagram. Only 6 (9%) children in the A‐FFIP and 9 (14%) children in the EIAU group were lost to follow‐up at T6.

### Sample characteristics

Baseline characteristics of the children and parents/family are shown in Table 1 and Appendix S2: Tables S1 and S2. At baseline, children showed a mean age of 49 (*SD* 10) months, a mean developmental age of 23.3 (*SD* 13.6) months and 26 (19.4%) were female. The ADI‐R toddler algorithm was used to support diagnosis in *n* = 31 (46%) of the A‐FFIP and *n* = 33 (50%) of the EIAU group. Parental education status was at a mean of 2.7 (*SD* 1.0) with a range of 0.5 to 5, and 9 (6.7%) had single custody. Eighty (60%) families included at least one parent who had immigrated to Germany as an adult. In the EIAU group, a higher rate of monolingual German children was observed, and developmental age as well as language development was slightly higher than in the A‐FFIP group. In addition to A‐FFIP/EIAU, >70% of the children in both groups received additional psychosocial interventions at baseline, such as speech and language or occupational therapy as well as individual support at kindergarten (see Table 1), which increased in the EIAU group to >90% over the course of the study (Appendix S2: Table S4). The average weekly intensity of additional interventions besides early intervention and integrative care at kindergarten between T1 and T6 was 1.6 hr/week (*SD* 1.0), and of integrative care 10.6 hr/week (*SD* 13.8), not differing between groups (Table 1). Primary and secondary endpoints did not differ between groups at baseline.

**Table 1:** Characteristics of the included children with ASD at baseline

	A‐FFIP (*N* = 68)	EIAU (*N* = 66)
Sex [female] *n* (%)	13 (19.1)	13 (19.7)
Age [months], mean (*SD*)	50.0 (10.0)	49.0 (10.0)
Developmental age [months], mean (*SD*)	21.6 (11.1) *n* = 64	25.1 (15.6) *n* = 62
Language development [months], mean (*SD*)	20.3 (14.5)	23.5 (19.0) *n* = 63
Full‐scale IQ/DQ, mean (*SD*)	45.8 (24.4) *n* = 64	52.0 (28.7) *n* = 62
Non‐verbal IQ/DQ, mean (*SD*)	58.9 (19.4)	63.8 (22.2)
Verbal IQ/DQ, mean (*SD*)	40.1 (28.7) *n* = 64	46.7 (34.2) *n* = 62
Out of home care at T1, *n* (%)	52 (76.5%)	52 (78.8%)
Any additional psychosocial intervention^a^ besides A‐FFIP/EIAU at T1, *n* (%)	49 (72.1%)	51 (77.3%)
Amount of additional psychosocial interventions without integrative care between T1 and T6; mean hours/week (*SD*)	1.6 (1.1)	1.7 (0.9)
Amount of integrative care at Kindergarten between T1 and T6; mean hours/week (*SD*)	10.7 (14.9)	10.4 (12.6)
Any medication at T1, *n* (%)	8 (11.8%)	9 (13.6%)
Single parenting with sole custody, *n* (%)	1 (1.5%)	8 (12.1%)
Parental education status (ISCED), mean (*SD*)	2.8 (0.9)	2.5 (1.0)
Parental immigration status, *n* (%)	43 (66.2%)	37 (58.7%)
German as only language spoken in family, *n* (%)	19 (29.2%)	26 (41.3%)
Sibling in family [yes] *n* (%)	46 (67.6%)	40 (60.6%)
BOSCC‐total score, mean (*SD*)	30.6 (10.2)	31.0 (10.2)
BOSCC social affect, mean (*SD*)	21.2 (8.0)	21.6 (7.8)
BOSCC repetitive behaviour, mean (*SD*)	9.3 (3.6)	9.4 (3.6)
ADOS‐2 calibrated severity score, mean (*SD*)	7.0 (1.6)	7.6 ± 1.6
ADOS‐2 social affect, mean (*SD*)	6.5 (1.8)	7.4 (1.6)
ADOS‐2 repetitive behaviour, mean (*SD*)	8.0 (1.5)	8.1 (1.4)
SRS16 parent, mean (*SD*)	28.8 (7.7) *n* = 65	27.1 (7.6) *n* = 56
RBS‐R parent total score, mean (*SD*)	32.4 (19.4) *n* = 64	34.3 (22.8) *n* = 55
RBS‐R parent insistence on sameness, mean (*SD*)	16.8 (10.5) *n* = 65	16.8 (11.6) *n* = 54
RBS‐R parent stereotyped behaviour, mean (*SD*)	8.6 (5.4) *n* = 64	9.7 (6.3) *n* = 56
RBS‐R parent self‐injury, mean (*SD*)	1.6 (2.2) *n* = 67	2.2 (4.2) *n* = 56
RBS‐R parent compulsive behaviour, mean (*SD*)	5.0 (4.9) *n* = 65	5.3 (5.4) *n* = 55
CBCL parent emotional reactivity, mean (*SD*)	5.5 (4.0) *n* = 66	5.3 (4.1) *n* = 54
CBCL parent anxious‐depressed, mean (*SD*)	4.4 (3.1) *n* = 67	4.2 (2.8) *n* = 56
CBCL parent somatic problems, mean (*SD*)	3.2 (2.6) *n* = 67	3.3 (3.0) *n* = 55
CBCL parent social withdrawal, mean (*SD*)	8.3 (3.0) *n* = 66	8.0 (3.3) *n* = 55
CBCL parent attention problems, mean (*SD*)	5.2 (2.1) *n* = 67	5.3 (1.9) *n* = 56
CBCL parent aggressive behaviour, mean (*SD*)	16.5 (7.2) *n* = 66	16.2 (7.3) *n* = 56
CBCL parent sleeping problems, mean (*SD*)	4.6 (3.3) *n* = 67	4.6 (3.3) *n* = 56
BRIEF‐P parent inhibitory self‐control, mean (*SD*)	26.7 (10.6) *n* = 64	27.6 (10.8) *n* = 53
BRIEF‐P parent flexibility, mean (*SD*)	18.5 (9.2) *n* = 64	18.8 (8.7) *n* = 54
BRIEF‐P parent emerging meta‐cognition, mean (*SD*)	27.4 (8.8) *n* = 58	25.9 (11.5) *n* = 52

ADOS‐2, Autism Diagnostic Observation Schedule – version 2; A‐FFIP, intervention group; BOSCC, Brief Observation of Social Communication Change; BRIEF‐P, Behaviour Rating Inventory of Executive Function – Preschool Version; CBCL, Child Behaviour Checklist; DQ, developmental quotient; EIAU, early intervention as usual; ESCS, Early Social Communication Scales; IQ, intelligence quotient; *n*, number; RBS‐R, Repetitive Behaviour Scale‐revised; *SD*, standard deviation; SRS16, Social Responsiveness Scale, 16 item version.

^a^ Psychosocial intervention besides A‐FFIP/EIAU includes music‐based, occupational, language or animal‐assisted intervention, special education/care at kindergarten (for details, see Tables S4 and S5 in Appendix S2).

### Impact of the SARS‐CoV‐2 pandemic

During the course of the study, the SARS‐CoV‐2 pandemic started in 2020. In Table 2, pandemic‐related information on both groups is shown. Over both groups, for 54 (40%) of the children, the intervention had to be stopped for 7–15 weeks due to the lockdowns implemented by the different regional governments at the beginning of the pandemic. In addition, due to strict mask‐wearing rules, a mean of 59% (*SD* 44) of all individual A‐FFIP intervention sessions had to be provided by therapists wearing masks. No specific information on the EIAU group is available on mask use during the intervention; still, pandemic restrictions were much stronger at University Hospitals, where A‐FFIP was provided compared with community‐based intervention centres, which provided EIAU. Also, the primary outcome BOSCC‐total as well as the direct observation secondary outcome measures were obtained by blinded, mask‐wearing testers during the pandemic. No effect of the mask worn by the tester was found comparing BOSCC‐total prior to intervention (at T2) without (mean 31.0, *SD* 10.2, *n* = 83) or with mask (mean 30.5, *SD* 10.2, *n* = 51; *p* = .935).

**Table 2:** SARS‐CoV‐2 pandemic‐related information

	A‐FFIP (*N* = 68)	EIAU (*N* = 66)
Interruption of treatment due to the SARS‐CoV‐2 pandemic, *n* (%)	Yes: 26 (38.2)	Yes: 28 (42.4)
Interruption of treatment in weeks, *n* (%)	0 weeks: 42 (61.8) 7 weeks: 15 (22.1) 15 weeks: 11 (16.2)	0 weeks 38 (57.6) 7 weeks 18 (27.3) 15 weeks 10 (15.2)
Use of masks during intervention, percentage of all intervention sessions, mean (*SD*)	59% (44)	Missing information
Use of masks during BOSCC testing at T2, *n* (%)	Yes: 27 (39.7)	Yes: 24 (36.4)
Use of masks during BOSCC testing at T4, *n* (%)	Yes: 41 (64.1) *N* = 64	Yes: 37 (61.7) *N* = 60
Use of masks during BOSCC testing at T6, *n* (%)	Yes: 52 (82.5) *N* = 63	Yes: 46 (82.1) *N* = 56

A‐FFIP, intervention group; EIAU, early intervention as usual; *N*, number.

### Intervention‐related characteristics

Intervention‐related characteristics are found in Table 3. A‐FFIP was provided for approximately 1 year. The absolute and relative weekly number of sessions was lower than the two planned sessions per week. Caregivers participated in approximately one‐third of the sessions, and, on average, one visit at kindergarten was possible. Self‐reported parental adherence to practice at home (mean 13.7, *SD* 2.1; possible maximum 20) and self‐reported parental competence (mean 7.3, *SD* 1.6; possible maximum 10) were both moderate throughout the intervention. Most of the children in the EIAU group did not receive any unspecific or ASD‐related early intervention (41/66, 62.1%), but a high rate of additional psychosocial interventions (Table 1; Appendix S2: Table S4). If available, the duration and number of sessions of EIAU showed a higher variability compared with the A‐FFIP group (Table 3).

**Table 3:** A‐FFIP and EIAU intervention characteristics between T2 and T6

	A‐FFIP (*N* = 66)	EIAU (*N* = 66)
Duration [weeks]; mean (*SD*)	53 (11) range 0–69	15 (24) range 0–69
Absolute number of intervention sessions, *n* (%)	72 (17) range 1–98	19 (32) range 0–138
Average weekly hours of intervention sessions; mean (*SD*)	1.38 (0.37) range 0.6–3.5	1 (1) range 0–10
Use of masks during intervention; mean percentage of all intervention sessions (*SD*)	59 (44)	n.a.
Absolute number of A‐FFIP sessions involving caregivers, *n* (%)	22 (18) range 1–84 2 missing	n.a.
Absolute number of A‐FFIP sessions involving kindergarten teachers at kindergarten, *n* (%)	1 (1) range 0–3 2 missing	n.a.

A‐FFIP, intervention group; EIAU, early intervention as usual; *N*, number; n.a., not applicable or not known; *SD*, standard deviation.

### Primary outcome

**Figure 2:**
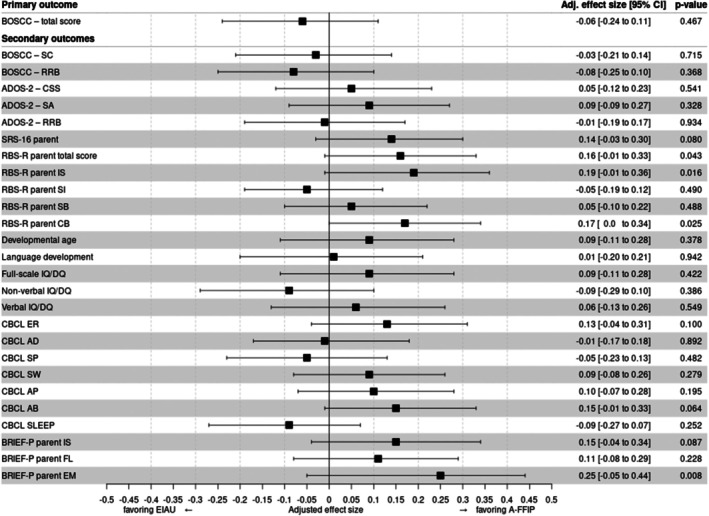
Forrest plot of effect sizes of the primary and selected secondary outcome measures. The effect size's confidence interval was obtained via bootstrap and may hence not be fully coherent with the *p*‐value. Results were obtained from the primary analysis set, that is, children with BOSCC measure at T2 (start), and at least one BOSCC measure at T4 (6 months) or T6 (12 months). AB, aggressive behaviour; AD, anxious‐depressed; ADOS‐2, Autism Diagnostic Observation Schedule – version 2, (sub‐)scales; A‐FFIP, intervention group; AP, attention problems; BOSCC, Brief Observation of Social Communication Change, subscales; BRIEF‐P, Behaviour Rating Inventory of Executive Function – Preschool Version, subscales; CB, compulsive behaviour; CBCL, Child Behaviour Checklist, subscales; CSS, calibrated severity score; DQ, developmental quotient; EIAU, early intervention as usual; EM, emerging meta‐cognition; ER, emotional reactivity; FL, flexibility; IQ, intelligence quotient; IS, inhibitory self‐control; IS, insistence on sameness; RRB, repetitive behavior; RSB‐R, Repetitive Behaviour Scale‐revised, subscales; SA, social affect; SB, stereotyped behaviour; SC, social communication; SI, self‐injury; SLEEP, sleeping problems; SP, somatic problems; SRS16, Social Responsiveness Scale, 16 item version; SW, social withdrawal

**Figure 3:**
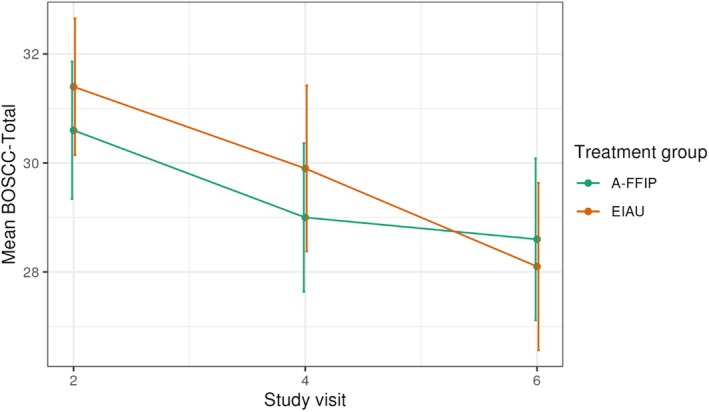
Time course of the BOSCC‐total by group in the primary analysis set. BOSCC, Brief Observation of Social Communication Change

The prespecified primary outcome BOSCC‐total at T6 was analyzed in the PAS. The children excluded from PAS (A‐FFIP *n* = 4, EIAU *n* = 6) were slightly younger (mean age 43 months, *SD* 8.0) and showed a slightly higher BOSCC‐total (mean 28.9, *SD* 9.1), a comparable developmental age (mean 24.4 months, *SD* 15.2) but slightly higher non‐verbal (mean 73.8, *SD* 24.7) and verbal IQ (mean 50.6, *SD* 39.6) compared with the children included in the FAS. In ITT analysis, no effect of A‐FFIP versus EIAU was observed (Figures 2 and 3, Table 4; Appendix S2, Tables S7 and S8; adjusted ES −0.06, 95% CI −0.24 to 0.11; *p* = .467; adjusted difference at T6–0.75, *SE* 1.03). Similar results were obtained in sensitivity analyses with discrete measurement times (Appendix S2, Table S9) and complete case analysis (Appendix S2, Table S13). A strong effect of treatment interruption due to the SARS‐CoV‐2 pandemic across both groups was observed (*p* = .01; Appendix S2, Table S10), with higher T6 scores and less improvement in children who had experienced the interruption. Across both, A‐FFIP and EIAU, the BOSCC‐total improved over time. The number of intervention sessions did not influence results across both groups (Appendix S2, Table S11). PP analysis on the respective subsample of A‐FFIP (*n* = 27) and EIAU (*n* = 54; reasons for exclusion Appendix S2, Table S6) gave essentially the same results (PP ES 0.05, 95% CI −0.17 to 0.28).

**Table 4:** Primary outcome and parent‐rated secondary outcomes in the full analysis set: Unadjusted T2, T4 and T6 measures

Outcome	A‐FFIP T1/2	EIAU T2	A‐FFIP T4	EIAU T4	A‐FFIP T6	EIAU T6
	(mean, *SD*, missing)	(mean, *SD*, missing)	(mean, *SD*, missing)	(mean, *SD*, missing)	(mean, *SD*, missing)	(mean, *SD*, missing)
BOSCC‐total score	30.6 (10.2)	31.0 (10.2)	29.0 (10.9) 4	29.9 (11.8) 6	28.6 (11.8) 5	28.1 (11.5) 10
BOSCC – SC	21.2 (8.0)	21.6 (7.8)	19.2 (8.2) 4	19.8 (9.5) 6	18.7 (8.7) 5	18.7 (8.9) 10
BOSCC – RRB	9.3 (3.6)	9.4 (3.6)	9.9 (3.8) 4	10.1 (3.9) 6	9.8 (4.3) 5	9.4 (4.0) 10
ADOS‐2 – CSS	7.0 (1.6)	7.6 (1.6)	7.2 (1.4) 4	7.4 (1.6) 8	6.9 (1.7) 7	7.4 (1.6) 12
ADOS‐2 – SA	6.5 (1.8)	7.4 (1.6)	6.5 (1.7) 4	6.9 (1.9) 8	6.2 (1.9) 7	6.9 (1.8) 12
ADOS‐2 –RRB	8.0 (1.5)	8.1 (1.4)	8.3 (1.2) 4	8.3 (1.3) 8	8.3 (1.3) 7	8.3 (1.3) 12
SRS16 parent	28.8 (7.7) 3	27.1 (7.6) 10	26.5 (8.6) 6	28.1 (7.9) 15	25.9 (8.9) 9	27.8 (8.0) 12
RBS‐R parent total score	32.4 (19.4) 4	34.3 (22.8) 11	30.9 (19.2) 5	36.1 (23.5) 14	29.2 (18.7) 9	38.3 (24.1) 12
RBS‐R parent IS	16.8 (10.5) 3	16.8 (11.6) 12	16.0 (10.1) 5	19.1 (12.3) 13	15.5 (10.2) 9	19.9 (11.9) 13
RBS‐R parent SI	1.6 (2.2) 1	2.2 (4.2) 10	1.9 (2.6) 5	2.3 (2.7) 13	1.8 (2.5) 8	2.6 (3.7) 12
RBS‐R parent SB	8.6 (5.4) 4	9.7 (6.3) 10	8.1 (5.2) 5	9.3 (6.8) 14	7.5 (5.1) 9	9.5 (6.7) 12
RBS‐R parent CB	5.0 (4.9) 3	5.3 (5.4) 11	4.9 (4.4) 5	6.1 (5.8) 13	4.5 (4.0) 8	6.2 (5.7) 12
Developmental age [months]	21.7 (11.2) 5	25.2 (15.8) 6	–	–	29.6 (17.1) 19	33.6 (20.5) 22
Non‐verbal IQ/DQ	58.7 (19.5) 1	62.6 (22.0) 3	–	–	58.8 (27.0) 12	63.5 (27.6) 19
Verbal IQ/DQ	45.9 (24.6) 5	52.1 (29.1) 6	–	–	48.4 (29.8) 19	52.2 (30.1) 22
CBCL ER	5.5 (4.0) 2	5.3 (4.1) 12	4.3 (3.8) 5	5.1 (3.6) 14	4.4 (3.8) 8	5.5 (3.7) 12
CBCL AD	4.4 (3.1) 1	4.2 (2.8) 10	4.1 (3.2) 5	4.4 (3.1) 13	4.3 (3.2) 8	4.4 (3.3) 12
CBCL SP	3.2 (2.6) 1	3.3 (3.0) 11	3.0 (2.8) 5	2.8 (2.2) 16	2.8 (2.3) 8	2.9 (2.1) 14
CBCL SW	8.3 (3.0) 2	8.0 (3.3) 11	7.4 (3.0) 5	7.6 (2.9) 13	7.0 (2.9) 8	7.6 (3.0) 12
CBCL AP	5.2 (2.1) 1	5.3 (1.9) 10	4.9 (2.3) 6	5.2 (2.0) 13	4.4 (2.1) 8	5.3 (2.4) 15
CBCL AB	16.5 (7.2) 2	16.2 (7.3) 10	14.5 (7.9) 5	17.7 (7.3) 13	14.5 (8.2) 8	16.6 (7.5) 12
CBCL SLEEP	4.6 (3.3) 1	4.6 (3.3) 10	4.0 (3.3) 5	4.2 (3.0) 14	4.3 (3.4) 8	3.8 (3.1) 12
BRIEF‐P parent IS	26.7 (10.6) 4	27.6 (10.8) 13	26.6 (11.5) 5	29.9 (10.0) 15	27.2 (12.0) 9	30.9 (11.3) 13
BRIEF‐P parent FL	18.5 (9.2) 4	18.8 (8.7) 12	17.9 (9.7) 5	19.9 (8.6) 14	18.7 (9.6) 9	20.8 (9.2) 12
BRIEF‐P parent EM	27.4 (8.8) 10	25.9 (11.5) 14	26.8 (11.1) 6	29.0 (11.6) 18	26.2 (11.8) 9	30.6 (11.9) 15

The data shown in this table refer to the full analysis set of A‐FFIP (*n* = 68) and EIAU (*n* = 66). AB, aggressive behaviour; AD, anxious‐depressed; ADOS‐2, Autism Diagnostic Observation Schedule – version 2, (sub‐)scales; A‐FFIP, intervention group; AP, attention problems; BOSCC, Brief Observation of Social Communication Change, subscales; BRIEF‐P, Behaviour Rating Inventory of Executive Function – Preschool Version, subscales; CB, compulsive behaviour; CBCL, Child Behaviour Checklist, subscales; CSS, calibrated severity score; DQ, developmental quotient; EIAU, early intervention as usual; EM, emerging meta‐cognition; ER, emotional reactivity; FL, flexibility; IQ, intelligence quotient; IS, inhibitory self‐control; IS, insistence on sameness; *n*, number; RRB, repetitive behavior; RRB, repetitive behavior; RSB‐R, Repetitive Behaviour Scale‐revised, subscales; SA, social affect; SB, stereotyped behaviour; SC, social communication; *SD*, standard deviation; SI, self‐injury; SLEEP, sleeping problems; SP, somatic problems; SRS16, Social Responsiveness Scale, 16 item version; SW, social withdrawal.

### Secondary outcomes

Secondary outcomes (Figure 2, Table 4; Appendix S2, Tables S7, S14–S17) suggest stronger improvements by A‐FFIP (*p*_all_ < .05) in stereotyped behaviour (parents: RBS‐R total score adjusted ES 0.16, 95% CI −0.01 to 0.33; IS adjusted ES 0.19, 95% CI −0.01 to 0.36; CB adjusted ES 0.17, 95% CI 0.0 to 0.34), executive functioning (BRIEF parents: EM adjusted ES 0.25, 95% CI −0.05 to 0.44; teachers: FL adjusted ES 0.21, 95% CI −0.01 to 0.45) and somatic problems (teachers: C‐TRF SP adjusted ES 0.27, 95% CI −0.07 to 0.50). Parent‐ and family‐related measures did not differ between groups.

### Moderation analyses

Of the baseline values age, verbal and non‐verbal IQ/DQ, sex, BOSCC‐total, SRS‐16, RBS‐R, parental education and migration status, only age showed a moderating effect on the primary outcome BOSCC‐total at T6, which differed between groups (Appendix S2, Table S18). In EIAU, older children showed slightly less improvement than younger ones (age × time: *p* = .059, *β* = 0.01, *SE* <0.01; age: *p* = .081, *β* = −0.26, *SE* 0.15). This age effect was not observed for A‐FFIP (age × time *p* = .687, age *p* = .251). A higher BOSCC‐total at T2 (*p* < .0001, *β* = 1.1, *SE* 0.14) correlated positively, and a higher full scale (*p* = .031, *β* = −0.12, *SE* 0.06) or verbal IQ (*p* = .025, *β* = −0.10, *SE* 0.05) negatively with BOSCC‐total at T6 across groups. An interaction with time, independent of group status, was found for RBS‐IS (parent: *p* = .036, *β* = −0.04, *SE* 0.18). Education or migration status as well as parental adherence did not show any main or interaction effects on the primary outcome.

### Safety

Adverse (AE) and serious adverse events (SAE) are shown in Table 5. SAE rarely occurred in both groups and were mainly related to hospital admissions. Infectious diseases (44.8%) were the most frequent AEs, followed by aggressive behaviour (13.4%; descriptively higher in EIAU), sleeping problems (9.8%; descriptively higher in A‐FFIP) and selective eating (4.9%; descriptively higher in EIAU). In the A‐FFIP group, 3 AEs were coded as possibly (infectious disease, aggressive behaviour) or certainly (injury) related to intervention.

**Table 5:** Adverse events

	A‐FFIP	EIAU	Total
	*N* (%)	*N* (%)	*N* (%)
Adverse events			
None	14 (20.6%)	22 (33.3%)	36 (26.9%)
Any [95% CI]	54 (79.4%) [0.684; 0.873]	44 (66.7%) [0.547; 0.768]	98 (73.1%) [0.651; 0.799]
Serious adverse events			
None	231 (98.7%)	151 (98.1%)	382 (98.5%)
Any	3 (1.3%)	3 (1.9%)	6 (1.5%)
Severity of adverse events			
Slight	174 (74.4%)	120 (77.9%)	294 (75.8%)
Moderate	55 (23.5%)	32 (20.8%)	87 (22.4%)
Severe	5 (2.1%)	2 (1.3%)	7 (1.8%)
Relation to intervention			
Definitely not	181 (77.4%)	153 (99.4%)	334 (86.1%)
Unlikely	50 (21.4%)	1 (0.6%)	51 (13.1%)
Possible	2 (0.9%)	0 (0.0%)	2 (0.5%)
Certain	1 (0.4%)	0 (0.0%)	1 (0.3%)
AE categories			
Aggressive behaviour	26 (11.1%)	26 (16.9%)	52 (13.4%)
Allergic reaction	1 (0.4%)	2 (1.3%)	3 (0.8%)
Anxiety	10 (4.3%)	5 (3.2%)	15 (3.9%)
Attention problems	0 (0.0%)	1 (0.6%)	1 (0.3%)
Autoaggressive behaviour	7 (3.0%)	5 (3.2%)	12 (3.1%)
Echolalia	1 (0.4%)	0 (0.0%)	1 (0.3%)
Elimination problems	2 (0.9%)	0 (0.0%)	2 (0.5%)
Hyperactivity	5 (2.1%)	3 (1.9%)	8 (2.1%)
Infectious disease	110 (47.0%)	64 (41.6%)	174 (44.8%)
Injury	14 (6.0%)	5 (3.2%)	19 (4.9%)
Loss of language	1 (0.4%)	0 (0.0%)	1 (0.3%)
Mannerism	1 (0.4%)	1 (0.6%)	2 (0.5%)
Noise sensitivity	2 (0.9%)	2 (1.3%)	4 (1.0%)
Pain	3 (1.3%)	3 (1.9%)	6 (1.5%)
Risky behaviours	2 (0.9%)	3 (1.9%)	5 (1.3%)
Seizures	1 (0.4%)	1 (0.6%)	2 (0.5%)
Selective eating	9 (3.8%)	10 (6.5%)	19 (4.9%)
Sleeping problems	25 (10.7%)	13 (8.4%)	38 (9.8%)
Stereotyped behaviour	3 (1.3%)	1 (0.6%)	4 (1.0%)
Tics	1 (0.4%)	2 (1.3%)	3 (0.8%)
Unusual behaviour	3 (1.3%)	1 (0.6%)	4 (1.0%)
Other	7 (3.0%)	6 (3.9%)	13 (3.4%)

Data on the full analysis set are reported. *N*, number. Adverse events were documented over 1 year; the percentage refers to the summary of all adverse events over the course of the trial by group.

## Discussion

The present A‐FFIP study is the first German multicentre RCT on toddlers and preschoolers with ASD implementing a complex, therapist‐provided NDBI with parent and kindergarten‐teacher training components in comparison with EIAU, namely the manualised A‐FFIP intervention. Regarding setting and weekly intervention amount, the A‐FFIP program fits well within the current framework of the public health care and social welfare system in Germany. The majority of all children received supplementary psychosocial interventions and integrative care during their time in kindergarten, reflecting the typical standard of preschool care for ASD in Germany, where ASD‐specific early intervention is relatively uncommon (Salomone et al., 2016).

The study has multiple strengths. The *study sample* can be considered as overall representative of the German population of preschool‐aged children with ASD and their families (https://www‐genesis.destatis.de/genesis/online), indicated by parental migration status, languages spoken at home, parental education and rural vs urban place of living. Both groups did not differ regarding baseline measures. Similar to other studies in pre‐schoolers (Sandbank et al., 2023), children showed below‐average developmental age at baseline, characteristic of early diagnosed children with ASD. In relation to *study design*, we chose the rater‐blinded direct behavioural measure BOSCC as the primary outcome. The BOSCC has only recently been used as an outcome in RCT (Green et al., 2022; Settanni et al., 2024; Swain et al., 2024). Similar to the blindly rated ADOS‐2, the BOSCC is a rater‐unbiased outcome for this age group. Highest *study quality* was ensured by regular monitoring, training and supervision of therapists within the study, and high inter‐rater reliability of the blinded BOSCC and ADOS‐2 raters.

Still, over 1 year of intervention, no difference between A‐FFIP and EIAU was observed in core ASD symptoms measured by the primary outcome BOSCC or the secondary outcome ADOS‐2. The amount of intervention over 1 year did not influence the primary outcome across groups, a finding corroborated by a recent meta‐analysis (Sandbank et al., 2024). Regarding social communication symptoms assessed by secondary outcomes, similarly no group differences were observed for the ADOS‐2‐CSS & ‐SA and parent‐rated SRS‐16, albeit the direction of the effect sizes was in favour of A‐FFIP for both outcomes.

During the course of the study, the SARS‐CoV2 pandemic severely impacted study execution and provision of intervention. It thus cannot be fully excluded that the validity and reliability of the primary outcome BOSCC and other direct observation secondary outcomes were influenced by mask wearing of testers; still, we did not observe differences in baseline BOSCC by the tester's mask use. The use of the BOSCC version for minimally verbal children (BOSCC‐MV) may also have impacted results. However, in a younger sample including minimally verbal children of three NDBIs, similarly, no intervention effects were observed by BOSCC‐MV (Swain et al., 2024), and the adapted BOSCC versions for older children did not detect any difference between groups in the PACT‐G trial (Green et al., 2022). Thus, it is unlikely that study results were influenced by BOSCC version or mask wearing of testers. By contrast, treatment interruption led to less improvement independent of intervention, probably reflecting the ongoing strain on families and children, especially at the start of the pandemic including the lockdowns (Isensee et al., 2022). As we did not obtain information on mask use in the EIAU group, we could not compare intervention effects by mask use of therapists. Still, it has to be assumed that especially social interaction and language learning were negatively affected by the far stricter mask use regulations in the A‐FFIP sample, provided through hospitals, compared with EIAU, provided primarily through early education institutions. For example, teaching imitation of facial movements was not any longer possible due to the masks. Also, the low parental participation in A‐FFIP intervention sessions and the lack of kindergarten visits for many children were mainly caused by pandemic regulations. Both aspects may also have negatively impacted A‐FFIP intervention outcome. The low number of A‐FFIP participants in the PP analysis is also explained by pandemic regulation effects.

In contrast to studies that applied the BOSCC to child–parent interactive play (Grzadzinski et al., 2016), we implemented the primary outcome BOSCC and the secondary outcome ADOS‐2 within a highly generalised, distant setting to the intervention, that is, a play session with an unknown, blinded interaction partner and new play material. This may have been a too complex task, as even typically developing preschoolers often show socially inhibited behaviour with strangers (Tan et al., 2024). Thus, sensitivity to measure change may have been reduced by our rigorous implementation of the BOSCC and the ADOS‐2. In contrast to our primary outcome, RCT on NDBIs with comparable weekly intervention amounts showing effects on social communication have used outcomes strongly matching their main intervention targets, such as the child's joint engagement (JASPER; parent‐delivered ESDM) or communication intentions (PACT) (Carruthers et al., 2020; Kasari et al., 2010; Waddington et al., 2024). Previous RCT on low‐ as well as high‐intensity NDBI or developmental interventions (DI) in ASD using the more distant ADOS‐2 CSS as an outcome did not observe any effects on core ASD symptoms over 1 year or less, similar to our study (ESDM: Fuller et al., 2020; Wang et al., 2022; PRT: Uljarević et al., 2021; PACT: Green et al., 2010). Follow‐up studies of these NDBI/DI over 2–6 years, by contrast, have reported stronger improvements in core ASD symptoms over time in the respective intervention groups (Estes et al., 2015; Pickles et al., 2016). Thus, we are currently conducting a longitudinal follow‐up of the A‐FFIP study sample testing long‐term effects and adding additional mediation analyses to explore if A‐FFIP will induce developmental cascades (Bednarz, Trapani, & Kana, 2020; Bradshaw et al., 2022). This is especially relevant, as moderation analysis found reduced effects of EIAU in older children. This finding was not observed for A‐FFIP, which showed similar effects across age. A‐FFIP thus may be a program fitting the needs of older pre‐schoolers with an established diagnosis of ASD, but not of very young children or children at risk for ASD (Waddington et al., 2024).

Our active control intervention, namely EIAU, may also have influenced findings on the primary and secondary outcomes. Children in EIAU received a slightly higher amount of psychosocial interventions than the A‐FFIP group, especially in the second 6 months of the 1‐year intervention. As the A‐FFIP manual has been published, and other early intervention manuals, such as the ESDM manual, are available in German, it is very likely that several of the children in the EIAU group did receive elements of ASD‐specific intervention through speech and language or occupational therapy. Also, the effect of behavioural interventions for pre‐schoolers often is smaller in high‐income countries compared with low‐ and middle‐income countries (Jeong et al., 2021), a finding which may be explained by the effects of the active control condition in high‐income countries.

Regarding parent‐rated repetitive behaviour, we observed small effects by A‐FFIP on overall RRB, IS as well as CB. This goes beyond most early intervention studies, which rarely assessed RRB or did not find group differences (Green et al., 2010). Still, given the large number of secondary outcomes, as well as the unblinded parent rating, this may have resulted in false positive findings, which thus need to be replicated by independent RCTs. Higher IS at baseline predicted stronger improvement over time across both groups on the primary outcome BOSCC, emphasising the need to study differential aspects of RRB in more detail in preschool RCTs in the future. In addition, we observed improved EF abilities reported by parents and teachers by A‐FFIP compared with EIAU, a finding that also requires replication. A‐FFIP allows for individualised, targeted intervention related to individual abilities, but also to challenging behaviours through a flexible implementation of effective behavioural intervention strategies, in addition to intervention techniques targeting social communication and play. If replicated, this may especially have impacted improved RRB and EF by A‐FFIP beyond EIAU. Moderation analysis did not observe any differential influence of the family's migration or education status on both interventions. Single custody could not be assessed as a moderator due to the low occurrence in both groups. Quality of life, parental stress and psychopathology, as well as adverse events, did not differ between groups. Taken together, these findings show that the A‐FFIP intervention is safe and feasible to be offered to all preschool children with ASD and their parents, irrespective of socio‐economic background.

Study limitations mainly pertain to the consequences of the SARS‐CoV2 pandemic, which were addressed by additional sensitivity analyses. As screening failures differed between sites, and descriptively some variable effects were observed between centres, characteristics of included children as well as study‐related procedures may also have differed between centres, despite intense monitoring, teaching and supervision throughout the study.

## Conclusion

A‐FFIP, a complex, low‐intensity NDBI, did not result in improved social communication symptoms over 1 year compared with EIAU, but small effects on improved core RRB symptoms and EF abilities were observed. Safety and feasibility indicate that A‐FFIP can be implemented in the standard early intervention setting in Germany. Similar to previous large‐scale RCT, 95% confidence intervals of effect sizes were large for all outcomes. This is indicative of subgroups of children with a differential sensitivity to environmental stimuli, such as early intervention (Cioni et al., 2016). Thus, future early intervention‐related research in ASD may aim at delineating subgroups of children sensitive to specific intervention techniques and proceed with adaptive intervention strategies (Kasari et al., 2025). In addition, therapist and parent‐implemented strategy use may be studied as a mediating variable of early intervention outcome (Brown et al., 2025).

## Declaration of interests

CMF and KT receive royalties for the A‐FFIP intervention manual. CMF, JG, TJ, JK, MN, VR and KT receive royalties for additional books on ASD, ADHD or MDD. CMF, JG, JK, MN, LP, VR, KT and RT received allowances for lectures on ASD and other mental health issues from public and private health care institutions. CMF has received consultation honoraria from the company IGES on a study evaluating staff regulations for standard mental health care in Germany (2024) and from the Institute for Quality and Efficiency in Health Care (Institut für Qualität und Wirtschaftlichkeit im Gesundheitswesen, IQWiG) for a brochure on parent and patient information on ASD (2023). VR carried out clinical trials in cooperation with Servier and Shire Pharmaceuticals/Takeda companies. The remaining authors have declared that they have no competing or potential conflicts of interest.

## Ethical information

This confirmatory phase‐III, prospective, randomised, multicentre, controlled, parallel‐group study with two treatment arms, an allocation ratio of 1:1 and six measurement time points was approved by the ethical committee of the medical faculty of Goethe University Frankfurt (4 April 2018; No. 10/18) and successively by the local ethical committees at Augsburg (26 June 2018; No. 18‐372), Dresden (21 January 2019; No. 41710218) and Wuerzburg (6 June 2018, No. 102/18_z‐sc). No major changes to the study protocol occurred after trial commencement. Study centres comprised the University Hospital Departments of Child and Adolescent Psychiatry, Psychosomatics and Psychotherapy at Frankfurt/Main (study‐coordinating site), Dresden and Wuerzburg, as well as Josefinum Augsburg, a general hospital specialised in Child and Adolescent Psychiatry and Psychotherapy. All four sites provide general mental health care to the entire local population, including specialised outpatient services for children with ASD. Independent data management and statistics were done by the Institute of Medical Biometry at Heidelberg University Hospital.

## Trial registration

The study has been preregistered with the German Clinical Trials Register (Deutsches Register Klinischer Studien, DRKS; ID: 00016330).

**Key points**

- A‐FFIP is a complex, low‐intensity naturalistic developmental behavioural intervention for toddlers and pre‐schoolers with ASD. Such interventions have rarely been studied by sufficiently powered, multicentre randomised‐controlled efficacy trials.
- Specific characteristics of the manualised A‐FFIP intervention are the individualised, flexible and complex, but still highly standardised approach targeting six core basic abilities and five developmental domains related to longitudinal development in ASD.
- A strong negative impact of the SARS‐CoV‐2 pandemic‐related regulations on the primary outcome was observed across both the A‐FFIP intervention and the active control early intervention as usual (EIAU).
- Regarding the blinded primary outcome, no effects on core ASD symptoms directly after the intervention were observed. Still, unblinded parent and teacher ratings resulted in improved repetitive behaviour and executive function measures.
- Given feasibility and safety, A‐FFIP is an NDBI for young children with ASD, which can easily be implemented within the German health care/social welfare system.

## Supporting information


**Appendix S1.** Supplementary material.

**Appendix S2.** Supplementary tables.

**Appendix S3.** Full list of staff.

## Data Availability

MK and LDS had full access to all the data in the study and take responsibility for the integrity of the data and the accuracy of the data analysis. Anonymised data will be made available to researchers or the journal editor upon request.
