# Supplementary material for: Complex, low‐intensity, individualised naturalistic developmental behavioural intervention in toddlers and pre‐schoolers with autism spectrum disorder: The multicentre, observer‐blind, parallel‐group randomised‐controlled A‐FFIP trial
Source: J Child Psychol Psychiatry. 2025 Mar 26;66(10):1500–13. doi: 10.1111/jcpp.14162 (PMC12447682; doi:10.1111/jcpp.14162)
Supplement: Supplementary file 1 — Appendix S1. Supplementary material. [file JCPP-66-1500-s002.pdf]

Supplementary material

Complex, low intensity, individualised naturalistic developmental behavioural intervention in toddlers and pre-schoolers with Autism Spectrum Disorder: The multicentre, observer-blind, parallel-group randomised controlled A-FFIP trial

Christine M. Freitag (1), Marietta Kirchner (2), Lukas D. Sauer (2), Solveig K. Kleber (1), Leonie Polzer (1), Naisan Raji (1), Christian Lemler (1), Ulrike Fröhlich (3), Julia Geissler (4), Melanie Ring (5), Veit Roessner (5), Regina Taurines (4), Michelle Noterdaeme (3), Karoline Teufel (1), Ziyon Kim (1)*, Janina Kitzerow-Cleven (1)*

* joint last authors

(1) Department of Child and Adolescent Psychiatry, Psychosomatics and Psychotherapy, Autism Research and Intervention Center of Excellence, University Hospital Frankfurt, Goethe-University Frankfurt am Main, Germany

(2) Institute of Medical Biometry, Heidelberg University Hospital, Heidelberg, Germany

(3) Josefinum, Department of Child and Adolescent Psychiatry, Psychosomatics and Psychotherapy, Augsburg, Germany

(4) Department of Child and Adolescent Psychiatry, Psychosomatics and Psychotherapy, Centre for Mental Health, University Hospital of Wuerzburg, 97080 Wuerzburg, Germany

(5) Department of Child and Adolescent Psychiatry, Technische Universität Dresden, 01307 Dresden, Germany

Table of contents

1. List of abbreviations 1
2. Detailed information on the Frankfurt early intervention program (A-FFIP) 2
3. A-FFIP Intervention targets checklist 4
4. Detailled information ethical votes and centres 5
5. Detailed information on assessment instruments 5
6. Detailed information on statistical methods 7
7. References 8

1. List of abbreviations

A-FFIP: Frankfurt Early Intervention Program for ASD

ADOS-2: Autism Diagnostic and Observation Schedule, version 2

BOSCC: Brief Observation for Communication Change

BRIEF-P: Behaviour Rating Inventory of Executive Function - Preschool Version

CBCL1½-5: Child Behaviour Checklist for Pre-schoolers

C-TRF: Kindergarten Teacher Report Form

DI: developmental intervention

EIAU: early intervention as usual

ISCED: International Standard Classification of Education

mv: minimally verbal

NDBI: Naturalistic developmental behavioural intervention

SRS: Social Responsiveness Scale

RBS-R: Repetitive Behaviour Scale-revised

RBS-R subscale IS: insistence on sameness

RBS-R subscale SB: stereotyped behaviour

RBS-R subscale SI: self-injury

RBS-R subscale CB: compulsive behaviour

2. Detailed information on the Frankfurt early intervention program (A-FFIP)

A-FFIP has been manualized as a comprehensive approach, which comprises evidence-based techniques and content, based on state-of-the-art developmental research in ASD. Developed within the funding limits of the German welfare system it is conceptualized as a low intensity program (2 hours/week over 2-3 years). The manual is standardized, giving many practical details and has been conceptualized allowing easy adoption in different intervention settings and care systems.

Intensity: A-FFIP comprises a maximum of 2 hours of intervention/week with a therapist and a co-therapist working with the child or – depending on individual abilities – with 2 children. Parents are invited to participate in at least every fifth session and are taught to use effective strategies for practising at home. For children attending kindergarten, psychoeducation of kindergarten-teachers is arranged three times/year.

Comprehensive approach

- A-FFIP targets the following six core basic abilities: (1) attentional control, (2) joint attention, (3) imitation, (4) representation, (5) planning and (6) self/other distinction,
- A-FFIP targets the following five developmental domains: (1) communication and language, (2) interaction and play, (3) emotion regulation, (4) cognition and (5) adaptive behaviour.

Individualized treatment targets: A-FFIP is an individualised intervention program providing specific intervention targets and methods based on the child's current ability level. The manual expects the therapists to choose exercises demanding slightly more than the child's current abilities to promote successful learning. Exercises are highly standardised as follows: For each of the core basic abilities and developmental domains, examples of exercises for beginners, intermediate and advanced learners are provided in the A-FFIP manual together with advice for therapists on how to correctly execute the exercises and to teach parents. Play material is used highly flexibly according to the preferences of the child, increasing the child's motivation to learn and to interact with the therapist. Based on results of a first standardised assessment phase and parental priority, a maximum of eight specific treatment targets is chosen in cooperation with the parents. Progress is monitored after each session by a standardised checklist included in the manual. Once the child has achieved a treatment target, a new target is focussed, which builds on the previous targets. Established skills are trained in an ongoing fashion during variable settings and situations to support generalization.

Intervention methods comprise effective, evidence based behavioural techniques supporting motivation and learning, and reducing interfering aggressive, anxious and stereotyped behaviour. They are applied during target-specific interactive play and teaching sessions, focussing on enhancing the child's coping and adaptive skills. The child's motivation to learn,

play, and socially interact is increased by natural reinforcement intrinsic to the child's interests and activities, and by consistent positive reinforcement of self-initiated learning.

- Standard behavioural, motivating techniques are used within natural social interaction to support self-initiated learning and play.
- Short- and long-term intervention targets are individually planed and practiced based on the current abilities of the child.
- Achievement of intervention targets is documented based on a detailed checklist of six core basic abilities and five developmental domains typically impaired in children with ASD. Each developmental domain comprises several developmental targets. Targets are practiced over three difficulty levels (beginner – intermediate – advanced).
- Two therapists work with the child to teach new skills, and to practice and generalize already acquired skills. While the main therapist is naturally interacting with the child and differentially reinforcing the child's initiations and reactions, the co-therapist works as a "shadow" behind the child directly helping to acquire new skills through individual and situational adequate prompting, shaping and fading techniques.
- Disruptive and stereotyped behaviour that interferes with learning processes is addressed by antecedent and consequence behaviour models focusing on enhancing the child's coping skills (e.g. by providing structure and routine), and by increasing interest and motivation to cooperate through natural reinforcement of the child's abilities.
- Anxious, obsessive-compulsive and selective eating behaviour is primarily addressed by structure and routines; if necessary due to symptom severity, gradual exposition training can also be done within the intervention.

Special features of the A-FFIP manual

- Detailed introduction on ASD specific compared to typical developmental course during infancy, toddlerhood and preschool age related to a broad range of cognitive, fine-motor, play, social interaction and language abilities.
- The manual provides a detailed checklist to document the individual's core basic abilities and developmental domain competencies, and provides related exercises for teaching and practicing these abilities and competencies over three levels (beginners, intermediate, advanced learners).
- All exercises are described in detail by providing explicit examples with information of necessary motivational material, prompting and possible natural re-inforcers. All exercises can (and should) be individually adapted regarding the child's interests and needs, which is encouraged in the manual by presenting ideas for variation.

Generalization of skills

- Parents are strongly involved in the intervention by joint decision on the individual child's therapy targets and regular practicing with the child during intervention sessions and at home.
- Examples for practicing new skills in daily situations are given for each target.
- Generalization of new skills is also encouraged by training of kindergarten teachers.
- Once the child is competent to socially interact with another child, the setting changes to 1x/week individual and 1x/week group-based intervention together with another child with ASD to practice peer play and interaction.

3. A-FFIP Intervention targets checklist

English adaptation from the German manual with the agreement of the authors © Springer Verlag: Teufel, K., Valerian, J., Wilker, C. & Freitag, C. (2017) *A-FFIP - Autismusspezifische Therapie im Vorschulalter*

	**Targets**	**Difficulty level***
Developmental targets are subdivided into 3 difficulty levels related to the child's current developmental status (beginners, intermediate, advanced learners). For each sub-target detailed exercises are provided, together with information on prompting, reinforcement, fading, examples for material and exercises for generalization during daily activities.		
**Core basic abilities**	Attentional control	Response to name
		Staying focused
		Flexible attention shifting
	Joint Attention I: Eye gaze following	Visually following an object
		Using eye contact during social interaction
		Pragmatic use of eye gaze
	Imitation	* Selected difficulty levels are provided as examples.<br><br>For more information and detailed exercises see: Teufel, K., Valerian, J., Wilker, C. & Freitag, C. (2017) *A-FFIP - Autismusspezifische Therapie im Vorschulalter.* Springer
	Representation	
	Planning	
	Self/other distinction	
**Developmental Domains**		
**Communication & Language**	Eye contact	
	Receptive vocabulary	
	Gestures	
	Initiation of language development (supported)	
	Expansion of expressive vocabulary	
	Asking and answering questions	
	Social interaction and communication	
**Social interaction & Play**	Joint Attention II: Reciprocity	
	Giving and exchanging	
	Sharing	
	Functional Play (non-interactive)	
	Basic interactive play	
	Interactive board games	
	Pretend Play	
	Role Play	
**Emotion regulation**	Emotion recognition	
	Emotion expression	
	Emotion regulation	
**Cognitive abilities**	Principles of matching I: Categories	
	Principles of matching II: Characteristics	
	Memorizing abilities	
	Drawing	
	Quantity understanding	
	Theory of mind	
	General knowledge	

**Developmental domains** (continued)		
**Adaptive behaviour**	Daily Routines	
	Toilet training	
	Problem solving	
	Managing dangerous situations	
	Spatial orientation	
	Temporal orientation	

4. Detailed information ethical votes and centres

This confirmatory phase-III, prospective, randomised, multi-centre, controlled, parallel-group study with two treatment arms, an allocation ratio of 1:1 and six measurement time points was approved by the ethical committee of the medical faculty of Goethe University Frankfurt (April 4th, 2018; No. 10/18), and successively by the local ethical committees at Augsburg (June 26th, 2018; No. 18-372), Dresden (January 21st, 2019; No. 41710218) and Wuerzburg (June 6th, 2018, No. 102/18_z-sc). No major changes to the study protocol occurred after trial commencement. Study centres comprised the University Hospital Departments of Child and Adolescent Psychiatry, Psychosomatics and Psychotherapy at Frankfurt/Main (study-co-ordinating site), Dresden and Wuerzburg as well as Josefinum Augsburg, a general hospital specialised in Child and Adolescent Psychiatry. All four sites provide general mental health care to the entire local population, including specialised outpatient services for children with ASD. Independent data management and statistics were done by the Institute of Medical Biometry at Heidelberg University Hospital.

5. Detailed information on assessment instruments

Video-based recordings of BOSCC, ADOS-2, and A-FFIP intervention sessions

Prior to obtaining any videos within the study, guidelines were developed on the standardised placement of the video cameras as well as on the respective settings and implemented materials for the different testing and intervention sessions. Site specific monitoring visits were done (JKC) to inspect the respective camera placements, testing environment, toys and play material, and to give feedback on the best implementation of the respective procedures each site.

Child: ASD-specific symptoms

ADI-R: The Autism Diagnostic Interview-revised is a semi-structured diagnostic interview with the parents, assessing the core autism symptoms in the areas of social interaction, communication, and stereotyped behaviour. In the present study, the ADI-R toddler algorithm was used for children < 4 years old (Bildt et al., 2015; Kim, Thurm, Shumway, & Lord, 2013), in children >= 4 years, the classical ADI-R algorithm was implemented (Bölte, Rühl, Schmötzer, & Poustka, 2008).

BOSCC: The implemented BOSCC version used within this trial is based on the research version of Dec 11th, 2017 (Grzadzinski et al., 2016; Kitzerow, Teufel, Wilker, & Freitag, 2016). It is the BOSCC version for minimally verbal children (Swain et al., 2024), implemented with an independent, unknown tester. A free-play situation of the child with the tester was video-recorded and analysed from video by blinded raters at Frankfurt. Pairwise inter-rater reliability for the BOSCC-total was 95.5% (95%-CI 95.3% to 95.7%), based on 70 patients and 140 ratings. The 140 ratings were randomly drawn from a total of 188 ratings to obtain exactly two ratings for each evaluated video. To these pairs of ratings, a linear model was fit to distinguish between variance explained by subjects and remaining variance. The free-play situation was highly standardised between the four study sites regarding the room environment as well as the implemented toys and play material. Prior to performing BOSCC

assessments with the children included into the RCT, all sites received a BOSCC implementation training, which was video-recorded, and feed-back was given to the testing staff at each site. The BOSCC consists of 15 items coded on a 6-point Likert scale ranging from 0 to 5. Items are coded from 2 x 5min of the child-tester play interaction session video based on a manualised decision tree. The social communication domain (SC) consists of 8 items (eye contact, facial expressions, gestures, vocalisations, integration of verbal and non-verbal communication, social overtures, engagement in activities). The repetitive behaviour domain (RRB) includes 4 items (sensory interests, manerisms, repetitive interests, stereotyped behaviour). The total score is the sum of the BOSCC – SC and –RRB domains.

ADOS-2: The ADOS-2 has been developed as diagnostic instrument, but has also been used as longitudinal measure of core ASD symptom severity based on direct behavioural observation (Szatmari et al., 2015). The ADOS-2 total comparison and domain calibrated severity scores (Lord, 2015) are calculated based on single items rated from standardised assessment videos (modules 1-3). Modules comprise a variable number of items related to social affect (SA) and restricted, repetitive behaviours (RRB). In the German version, internal consistency varied from α=0.87 to α=0.92 in the SC and from α=0.51 to α=0.66 in the RRB domain (Poustka et al., 2015). In the A-FFIP study, we established inter-rater reliability (IRR) for modules 1-3 based on 76 randomly selected videos of 128 videos, and observed a bias corrected IRR = 83.6 (95%-CI 79.5 – 86.9) for the ADOS total comparison score; ADOS-2 social affect: IRR = 85.9 (95%-CI 83.8 – 87.7), ADOS-2 repetitive behaviour IRR = 46.9 (95%-CI 19.8 – 67.3).

Parent/teacher-rated social responsiveness was assessed by the change-sensitive SRS-16 item version (Sturm, Kuhfeld, Kasari, & McCracken, 2017). The parent-rated short version has been shown to capture the range of the distribution of social responsiveness similarly well as the original long 65 item version in 3-5-year old children referred for evaluation of ASD (Lyall et al., 2022). The SRS-16 summary score shows an internal consistency of α=0.96.

Parent/teacher-rated RRB was assessed by the 43-item Repetitive Behaviour Scale-Revised (RBS-R) (Kästel et al., 2021). The four factors of the RBS-R, i.e. insistence on sameness (IS), self-injury (SI), stereotyped behaviour (SB) and compulsive behaviour (CB), show an internal consistency between α=0.75 and α=0.94, total score α=0.96.

Child: Cognition and language

Non-verbal and verbal cognitive development: No valid uniform IQ test covering the entire developmental age of the sample is available in Germany. Thus, developmental age equivalences and IQ/DQ were derived from the following standardized tests: Bayley Scales of Infant and Toddler Development – Third Edition (Bayley-III, German version with US-American norms) (Reuner, 2015) and the German version of the Wechsler Preschool and Primary Scale of Intelligence with German norms (WPPSI-III) (Petermann, 2014).

If children had a chronological age ≥ 36 months and were able to comprehend instructions, the WPPSI-III was applied. If children could not follow instructions of the WPPSI-III or had a chronological age < 36 months, the Bayley-III was obtained. In addition, if a child was able to comprehend the instructions of the WPPSI-III subscales related to performance IQ (PIQ) but did not comprehend the instructions of the WPSSI-III subscales for the general language composite (GLC), the Bayley-III scales for the verbal DQ and the WPPSI-III scale for the non-verbal IQ were applied.

The non-verbal IQ was calculated based on the Bayley-III as follows: Non-verbal IQ = developmental age (subscale cognition) / chronological age (month) x 100. Based on the WPPSI-III, the non-verbal IQ is equal to the performance IQ scale.

The verbal IQ was calculated based on the Bayley-III as follows: Verbal IQ = [developmental age (subscale receptive language + subscale expressive language)/2] / chronological age (month) x 100. Based on the WPPSI-III, the verbal IQ is equal to the verbal IQ scale.

The full scale IQ was calculated based on the Bayley-III as follows: Full-scale IQ = [developmental age (subscale cognition + subscale receptive language + subscale expressive language)/3] / chronological age (month) x 100. Based on the WPPSI-III, the full scale IQ is equal to the full-scale (total score) IQ.

If the nonverbal IQ was measured by the WPPSI-III and the verbal abilities by the Bayley-III, then the full-scale-IQ was calculated as follows: (Bayley-III based verbal IQ + WPPSI-III based non-verbal IQ)/2.

Developmental age equivalents (DA, months) were calculated as follows: Based on the Bayley-III, DA was calculated as the mean of the developmental age scores of the cognitive and language scales: DA = (subscale cognition + subscale receptive language + subscale expressive language)/3. Based on the WPPSI-III DA was calculated as follows: DA = chronological age (month) / 100*full-scale IQ.

If the nonverbal IQ was measured by the WPPSI-III and the verbal abilities by the Bayley-III, then the full-scale-IQ is calculated as follows: DA = (Bayley-III subscale receptive language + Bayley-III subscale expressive language + WPPSI-III based DA)/3.

Language development (LD) = receptive and expressive language skills [months] were calculated as follows: Based on the Bayley-III, LD was calculated as the mean of the developmental age scores of the language scales: DA = (subscale receptive language + subscale expressive language)/2. Based on the WPPSI-III LD was calculated as follows: LD = chronological age (month) / 100*General Language Scale IQ.

Child: Additional behavioural measures

The German version of the parent-rated Child Behaviour Checklist for Preschoolers (CBCL1½-5 - 100 items) and the corresponding kindergarten teacher-rated Teacher Report Form (C-TRF – 100 items) are valid and reliable internationally implemented scales, giving eight (C-TRF) respectively nine (CBCL1½-5) social-emotional or behavioural problem dimensions (Plück, Scholz, & Döpfner, 2022).

The German version of the 63-item Behaviour Rating Inventory of Executive Function-Preschool Version (BRIEF-P) is a rating scale assessing five dimensions of executive functioning (EF) in preschoolers. Internal consistency varies between α=.75 and α=.96 depending on rater/subscale (Daseking & Petermann, 2013).

Caregiver and family-related measures

Parenting efficacy was derived from the German adaptation of the Parenting Sense of Competence Scale (PSOC/FSW) (Kliem, Kessemeier, Heinrichs, Döpfner, & Hahlweg, 2014), for which with internal consistencies for mother and father ratings of α=.78 / .79) have been reported.

The Depression, Anxiety and Stress Scales – short form (DASS-21) (Nilges & Essau, 2015) were implemented to study stress-related, anxious and depressed symptoms in parents. The 3 dimensions show an internal consistency from α=.76 to α=.91. Convergent validity to other measures of depression and anxiety is good (r=.59 to r=.86).

The Family quality of Life Survey-2006 ID/DD version (FQOLS-2006-ID/DD) covers nine domains, of which four (family relationships, external support, leisure time, and community integration) were assessed in this study. A validation study reported high internal consistency (α=.85) and concurrent validity (r=.63) with another quality of life measure (Perry & Isaacs, 2015).

6. Detailed information on statistical methods

Descriptive analysis, primary and secondary analyses, moderator analysis and the safety analysis were described in the main text. The primary analysis was accompanied by several sensitivity analyses, investigating

- The inclusion of discrete visits instead of the continuous time of BOSCC-total measurement,
- The interaction of treatment and the number of attended therapy sessions (the exact number was known for the A-FFIP group, but only estimated for the EIAU group),
- The use of the PP set instead of the PAS,
- The use of complete cases only,
- The effect of the SARS-CoV-2 pandemic on the primary analysis by (i) including a covariate to adjust for the treatment interruption, (ii) separate MMRMs for patients with and without treatment interruption, (iii) separate MMRMs for all observations before the treatment interruption and all observations after the treatment interruption, (iv) separate MMRMs for patients whose sessions were affected by therapist mask use in all sessions, in some sessions and patients who were not affected by mask use (for the EIAU group, data on mask use was not available and we used mask use during BOSCC measurement as a proxy). Results of analysis number (iv) were not reported as the mask use during EIAU was less strict than during A-FFIP and during the BOSCC measurement sessions; hence, this sensitivity analysis was not deemed valid.

The trajectory analysis of parental adherence was performed by means of semiparametric group-based trajectory modelling with the PROC TRAJ macro in SAS. The aim was to identify clusters of individuals with similar trajectories (evolution of PATCS over time). Four types of PATCS trajectories were expected but finally, a three group solution was identified. The shapes of the trajectories were explored in the model by starting with third order (cubic) of parameter estimates. In the final model, we used the highest order which was significant for each trajectory.

Cluster analysis of parental adherence trajectories over the time of the intervention resulted in three groups with low (n=22: mean adherence 11.6, SD 0.8; mean competence 6.5, SD 1.7), medium (n=38: mean adherence 14.2, SD 1.3; mean competence 7.5, SD 1.4) and high (n=6: mean adherence 17.8, SD 1.2; mean competence 9.1, SD 0.5) adherence.

Inter-rater reliability (IRR) between raters of the video-coded assessments BOSCC, ADOS, DCMA and ESCS was calculated by drawing two random ratings for each video in the validation data set and then fitting the model $x_{ij}=\mu+b_j+\epsilon_{ij}$, where x_{ij} denotes the rating of the video j rated by one of the two randomly selected raters *i*, μ denotes the unknown true population mean score, b_j denotes the unknown difference between the subject's true score and the population mean, and ϵ_{ij} denotes the random deviation from the subject's true score that was introduced by the rater. IRR was then calculated as the intra-class correlation coefficient $\sqrt{((\sigma_b^2)/(\sigma_b^2+\sigma_\epsilon^2))}$, where σ_b^2 denotes the variance explained by the subjects and σ_ϵ^2 is the remaining variance of the random deviation (Shrout & Fleiss, 1979). Confidence intervals were obtained using the jack-knife method together with Fisher's Z-transformation (Feng, Svetnik, Coimbra, & Baumgartner, 2014).

7. References

Bildt, A. de, Sytema, S., Zander, E., Bölte, S., Sturm, H., Yirmiya, N., et al. (2015). Autism Diagnostic Interview-Revised (ADI-R) Algorithms for Toddlers and Young Preschoolers: Application in a Non-US Sample of 1,104 Children. *Journal of Autism and Developmental Disorders, 45*(7), 2076–2091.

Bölte, S., Rühl, D., Schmötzer, G., & Poustka, F. (2008). *ADI-R: Diagnostisches Interview für Autismus - Revidiert: Deutsche Fassung des Autism Diagnostic Interview - Revisted (ADI-R)* (1. Nachdruck). Bern: Huber.

Daseking, M., & Petermann, F. (2013). *BRIEF-P. Verhaltensinventar zur Beurteilung exekutiver Funktionen für das Kindergartenalter.* Göttingen: Hogrefe.

Feng, D., Svetnik, V., Coimbra, A., & Baumgartner, R. (2014). A comparison of confidence interval methods for the concordance correlation coefficient and intraclass correlation coefficient with small number of raters. *Journal of Biopharmaceutical Statistics, 24*(2), 272–293.

Grzadzinski, R., Carr, T., Colombi, C., McGuire, K., Dufek, S., Pickles, A., & Lord, C. (2016). Measuring Changes in Social Communication Behaviors: Preliminary Development of the Brief Observation of Social Communication Change (BOSCC), *Journal of Autism and Developmental Disorders 46*(7), 2464–2479.

Kästel, I. S., Vllasaliu, L., Wellnitz, S., Cholemkery, H., Freitag, C. M., & Bast, N. (2021). Repetitive Behavior in Children and Adolescents: Psychometric Properties of the German Version of the Repetitive Behavior Scale-Revised. *Journal of Autism and Developmental Disorders, 51*(4), 1224–1237.

Kim, S. H., Thurm, A., Shumway, S., & Lord, C. (2013). Multisite study of new autism diagnostic interview-revised (ADI-R) algorithms for toddlers and young preschoolers, *43*(7), 1527–1538.

Kitzerow, J., Teufel, K., Wilker, C., & Freitag, C. M. (2016). Using the brief observation of social communication change (BOSCC) to measure autism-specific development. *Autism Research, 9(9), 940–950.*

Kliem, S., Kessemeier, Y., Heinrichs, N., Döpfner, M., & Hahlweg, K. (2014). Der Fragebogen zur Selbstwirksamkeit in der Erziehung (FSW). *Diagnostica, 60*(1), 35–45.

Lord, C. (2015). *ADOS-2: Diagnostische Beobachtungsskala für autistische Störungen - 2: Manual.* Bern: Huber.

Lyall, K., Rando, J., Toroni, B., Ezeh, T., Constantino, J. N., Croen, L. A., et al. (2022). Examining shortened versions of the Social Responsiveness Scale for use in autism spectrum disorder prediction and as a quantitative trait measure: Results from a validation study of 3-5-year old children. *JCPP advances, 2*(4), e12106.

Nilges, P., & Essau, C. (2015). [Depression, anxiety and stress scales: DASS—A screening procedure not only for pain patients]. *Schmerz, 29*(6), 649–657.

Perry, A., & Isaacs, B. (2015). Validity of the Family Quality of Life Survey-2006, *28*(6), 584–588.

Petermann, F. (Ed.) (2014). *Always learning. Wechsler preschool and primary scale of intelligence- III: (WPPSI-III); Deutsche Version* (3., überarb. und erw. Aufl.). Frankfurt, M: Pearson.

Plück, J., Scholz, K.-K., & Döpfner, M. (2022). *CBCL/ 1 ½-5, C-TRF/ 1 ½-5: Deutsche Kleinkind- und Vorschulalter-Formen der Child behavior checklist von Thomas M. Achenbach und Leslie A. Rescorla: Elternfragebogen für Klein- und Vorschulkinder und Fragebogen für Erzieherinnen von Klein- und Vorschulkindern: Manual* (1. Auflage). Göttingen: Hogrefe.

Poustka, L., Rühl, D., Feineis-Matthews, S., Poustka, F., Hartung M, & Bölte, S. (Eds.) (2015). *Diagnostische Beobachtungsskala für Autistische Störungen - 2.* Bern: Huber.

Reuner, G. (Ed.) (2015). *Bayley Skales of Infant and Toddler development - third edition. German version.* Göttingen: Hogrefe.

Shrout, P. E., & Fleiss, J. L. (1979). Intraclass correlations: uses in assessing rater reliability. *Psychological Bulletin, 86(2*), 420–428.

Sturm, A., Kuhfeld, M., Kasari, C., & McCracken, J. T. (2017). Development and validation of an item response theory-based Social Responsiveness Scale short form. *Journal of Child Psychology and Psychiatry, 58*(9), 1053–1061.

Swain, D., Li, Y., Brown, H. R., Petkova, E., Lord, C., Rogers, S. J., et al. (2024). Implementing a Uniform Outcome Measurement Approach for Early Interventions of Autism Spectrum Disorders. *Journal of the American Academy of Child and Adolescent Psychiatry*, epub

Szatmari, P., Georgiades, S., Duku, E., Bennett, T. A., Bryson, S., Fombonne, E., et al. (2015). Developmental trajectories of symptom severity and adaptive functioning in an inception cohort of preschool children with autism spectrum disorder. *JAMA Psychiatry 72*(3), 276–283.
