# Supplementary material for: Complex, low‐intensity, individualised naturalistic developmental behavioural intervention in toddlers and pre‐schoolers with autism spectrum disorder: The multicentre, observer‐blind, parallel‐group randomised‐controlled A‐FFIP trial
Source: J Child Psychol Psychiatry. 2025 Mar 26;66(10):1500–13. doi: 10.1111/jcpp.14162 (PMC12447682; doi:10.1111/jcpp.14162)
Supplement: Supplementary file 2 — Appendix S2. Supplementary tables. [file JCPP-66-1500-s003.pdf]

Supplementary tables

Complex, low intensity, individualised naturalistic developmental behavioural intervention in toddlers and pre-schoolers with Autism Spectrum Disorder: The multicentre, observer-blind, parallel-group randomised-controlled A-FFIP trial

Christine M. Freitag (1), Marietta Kirchner (2), Lukas D. Sauer (2), Solveig K. Kleber (1), Leonie Polzer (1), Naisan Raji (1), Christian Lemler (1), Ulrike Fröhlich (3), Tomasz Jarczok (3), Julia Geissler (4), Franziska Radtke (4), Melanie Ring (5), Veit Roessner (5), Regina Taurines (4), Michelle Noterdaeme (3), Karoline Teufel (1), Ziyon Kim (1)*, Janina Kitzerow-Cleven (1)*
* joint last authors

Supplementary table 1: Characteristics of the included children at baseline – (kindergarten-) teacher reports

	A-FFIP (N=68)	EIAU (N=66)
SRS16 teacher, mean (SD), n	31.2 (9.5) n=50	31.2 (10.0) n=39
RBS-R teacher total score, mean (SD), n	30.9 (20.6) n=44	29.3 (19.7) n=36
RBS-R teacher insistence on sameness, mean (SD), n	15.4 (10.4) n=28	16.6 (10.8) n=25
RBS-R teacher stereotyped behaviour, mean (SD), n	8.8 (6.1) n=52	9.3 (6.1) n=43
RBS-R teacher self-injury, mean (SD), n	1.8 (2.3) n=53	2.0 (3.2) n=43
RBS-R teacher compulsive behaviour, mean (SD), n	4.1 (4.9) n=52	3.8 (3.9) n=41
CTRF teacher emotional reactivity, mean (SD), n	4.4 (3.0) n=50	4.0 (2.6) n=40
CTRF teacher anxious-depressed, mean (SD), n	3.7 (3.3) n=48	2.9 (2.8) n=41
CTRF teacher somatic problems, mean (SD), n	1.0 (1.2) n=48	1.0 (1.0) n=41
CTRF teacher social withdrawal, mean (SD), n	10.8 (4.4) n=49	10.4 (4.2) n=41
CTRF teacher attention problems, mean (SD), n	10.6 (4.0) n=52	11.3 (3.7) n=41
CTRF teacher aggressive behaviour, mean (SD), n	11.8 (5.4) n=48	12.1 (7.4) n=40
BRIEF-P teacher inhibitory self-control, mean (SD), n	27.8 (11.0) n=50	28.7 (12.7) n=40
BRIEF-P teacher flexibility index, mean (SD), n	19.7 (9.2) n=51	21.1 (9.4) n=39
BRIEF-P teacher emerging metacognition, mean (SD), n	28.1 (12.8) n=44	28.2 (12.9) n=34

Legend supplementary table 1:
A-FFIP=intervention group; EIAU=early intervention as usual,
BRIEF-P: Behaviour Rating Inventory of Executive Function - Preschool Version, CTRF: Preschool Children Teacher Report Form, RBS-R: Repetitive Behaviour Scale-revised, SRS16: Social Responsiveness Scale, 16 item version; n: number, SD: standard deviation.

Supplementary table 2: Family- and parent-related measures at baseline

	A-FFIP (N=68)	EIAU (N=66)
Family related quality of life, mean (SD), n	52.4 (10.3) n=67	48.7 (9.4) n=65
DCMA synchrony, mean (SD), n	64.3 (30.3)	63.2 (31.5)
DCMA communication, mean (SD), n	113.0 (29.9)	113.7 (34.9)
PSOC total score mother, mean (SD), n	18.2 (4.6) n=66	19.2 (4.0) n=53
PSOC total score father, mean (SD), n	17.0 (4.7) n=59	18.5 (4.2) n=48
DASS-21 depression mother, mean (SD), n	4.2 (4.0) n=66	4.1 (4.5) n=51
DASS-21 anxiety mother, mean (SD), n	2.2 (3.1) n=66	2.7 (4.0) n=52
DASS-21 stress mother, mean (SD), n	7.3 (5.1) n=66	7.1 (4.4) n=53
DASS-21 depression father, mean (SD), n	3.8 (4.6) n=61	2.5 (3.3) n=48
DASS-21 anxiety father, mean (SD), n	2.1 (3.6) n=61	1.8 (3.5) n=48
DASS-21 stress father, mean (SD), n	6.0 (4.8) n=61	5.5 (4.3) n=48

Legend supplementary table 2:
DASS-21: Depression, Anxiety and Stress Scales – short form, DCMA: Dyadic Communication Measure for Autism – Revised, PSOC: Parenting Sense of Competence; n: number, SD: standard deviation.

Supplementary table 3: Fidelity of A-FFIP therapists between T2 and T6 by study site

	Frankfurt	Augsburg	Dresden	Würzburg
Number of patients with coded therapy session videos (n)	33	15	6	11
Number of coded videos per patient - mean (SD)	11 (9)	18 (13)	28 (10)	19 (11)
Number of coded videos per patient – range	3, 37	8, 42	9, 34	9, 35
Fidelity of therapists - mean (SD)	1.94 (0.04)	1.91 (0.04)	1.92 (0.04)	1.89 (0.05)
Fidelity of therapists - range	1.83, 2.00	1.80, 1.96	1.86, 1.95	1.80, 1.97

Legend supplementary table 3: n: number, SD: standard deviation.

Supplementary table 4: Additional psychosocial and pharmacological interventions at all single measurement time-points T1 to T6

	A-FFIP (N=68)	EIAU (N=66)
Child is in out-of-home care at T1, n (%)	52 (76.5%)	52 (78.8%)
Out-of-home care at T1,<br>mean hours / day (SD), range	4.5 (3.1)<br>0 - 10	4.4 (2.8)<br>0 - 9
Any medication at T1, n (%)	8 (11.8%)	9 (13.6%)
Any additional psychosocial intervention* at T1, n (%)	49 (72.1%)	51 (77.3%)
Additional psychosocial interventions T1<br>mean hours / week (SD), range	8.9 (13.5)<br>0 - 44	7.0 (10.8)<br>0 - 34
Child is in out-of-home care at T2, n (%)	55 (80.9%)	52 (78.8%)
Out-of-home care at T2,<br>mean hours / day (SD), range	4.7 (2.9)<br>0 - 10	4.4 (2.8)<br>0 - 9
Any medication at T2, n (%)	8 (11.8%)	9 (13.6%)
Any additional psychosocial intervention* at T2, n (%)	49 (72.1%)	51 (77.3%)
Additional psychosocial interventions T2<br>mean hours / week (SD), range	9.4 (13.6)<br>0 – 44	7.0 (10.8)<br>0 - 34
Child is in out-of-home care at T3, n (%)	56 (84.8%) n=66	51 (82.3%) n=62
Out-of-home care at T3,<br>mean hours / day (SD), range	5.4 (3.0)<br>0 - 13	4.5 (2.7)<br>0 - 9
Any medication at T3, n (%)	12 (18.2%) n=66	12 (19.4%) n=62
Any additional psychosocial intervention* at T3, n (%)	55 (83.3%) n=66	55 (88.7%) n=62
Additional psychosocial interventions T3<br>mean hours / week (SD), range	10.5 (14.4)<br>0 - 44	8.7 (11.5)<br>0 - 41
Child is in out-of-home care at T4, n (%)	54 (84.4%) n=64	52 (86.7%) n=60
Out-of-home care at T4,<br>mean hours / day (SD), range	5.6 (3.5) n=64<br>0 - 18	5.0 (2.8) n=60<br>0 - 12
Any medication at T4, n (%)	13 (20.3%) n=64	13 (21.7%) n=60
Any additional psychosocial intervention* at T4, n (%)	54 (84.4%) n=64	59 (98.3%) n=60
Additional psychosocial interventions T4<br>mean hours / week (SD), range	11.8 (15.2)<br>0 - 45	11.6 (13.4)<br>0 - 51
Child is in out-of-home care at T5, n (%)	52 (82.5%) n=63	52 (92.9) n=56
Out-of-home care at T5,<br>mean hours / day (SD), range	5.8 (3.9) n=64<br>0 - 18	5.3 (2.8) n=60<br>0 -16
Any medication at T5, n (%)	15 (23.8%) n=63	9 (13.6%) n=56
Any additional psychosocial intervention* at T5, n (%)	50 (79.4%) n=63	54 (96.4%) n=56
Additional psychosocial interventions T5<br>mean hours / week (SD), range	10.3 (14.1)<br>0 - 45	12.1 (13.1)<br>0 - 51
Child is in out-of-home care at T6, n (%)	59 (93.7%) n=63	53 (93.0%) n=57
Out-of-home care at T6,<br>mean hours / day (SD), range	5.8 (2.8) n=63<br>0 - 18	5.9 (3.0) n=57<br>0 - 16

Any medication at T6, n (%)	15 (23.8%) n=63	12 (21.1%) n=57
Any additional psychosocial intervention* at T6, n (%)	56 (88.9%) n=63	52 (91.2%) n=57
Additional psychosocial interventions T6<br>mean hours / week (SD), range	12.8 (17.3)<br>0 - 88	13.5 (14.4)<br>0 - 51

Legend supplementary table 4:
* Additional psychosocial intervention besides A-FFIP/EIAU includes music-based, occupational, speech and language or animal-assisted intervention, special education/care or individual support at kindergarten
A-FFIP=intervention group; EIAU=early intervention as usual;
n: number; SD: standard deviation.

Supplementary table 5: Mean hours of additional interventions between T2 and T6

	A-FFIP (N=68)	EIAU (N=66)
Speech and language therapy n (%)<br>mean hours / week (SD)	n=48 (70.6)<br>0.90 (0.40)	n=45 (68.2)<br>0.98 (0.57)
Occupational therapy; n (%)<br>mean hours / week (SD)	n=40 (58.8)<br>0.80 (0.35)	n=48 (72.7)<br>0.87 (0.42)
Animal based intervention;<br>n (%), mean hours / week (SD)	n=6 (0.9)<br>0.62 (0.45)	n=5 (7.5)<br>0.60 (0.38)
Music based intervention n (%)<br>mean hours / week	0 (0.0)	1 (1.5)<br>0.70
Individual support at Kindergarten n (%)<br>mean hours / week (SD)	n=27 (39.7)<br>21.78 (9.88)	n=32 (48.5)<br>16.68 (10.16)

Legend supplementary table 5:
A-FFIP=intervention group; EIAU=early intervention as usual,
n: absolute number of children receiving intervention; % percentage;
SD: standard deviation.

Supplementary table 6: Reasons for exclusion of per-protocol analysis set

	A-FFIP<br>N=41/68 excluded	EIAU<br>N=12/66 excluded	Total<br>N=53/134 excluded
Major protocol violation #	1 (2.4%)	3 (25.0%)	4 (7.5%)
BOSCC T6 missing	5 (12.2%)	10 (83.3%)	15 (28.3%)
A-FFIP: <68 intervention sessions	22 (53.7%)	n.a.	22 (53.7%)
A-FFIP: No visit of therapist at kindergarten during intervention	23 (56.1%)	n.a.	23 (56.1%)
A-FFIP: Family attended <15% of all intervention sessions	14 (34.1%)	n.a.	14 (34.1%)

Legend supplementary table 6:
Major protocol violations A-FFIP: study drop out (n=1); EIAU: study drop-out (n=1), no teacher questionnaires at T4 distributed (n=1); direct testing of child at T6 not possible (n=1)
A-FFIP=intervention group; EIAU=early intervention as usual,
BOSCC: Brief Observation of Social Communication Change.

Supplementary table 7: Primary outcome and selected secondary outcomes in the primary analysis set

**Outcome**	**A-FFIP (PAS N=64) mean change T1/T2 to T6 (SD)**	**EIAU (PAS N=60) mean change T1/T2 to T6 (SD)**	**Adjus-ted effect size**	**95%-CI#**	**p-value**
BOSCC-total score	-2.1 (6.9) n=63	-2.9 (6.4) n=56	-0.06	-0.24 to 0.11	0.467
BOSCC – SC	-2.5 (6.0) n=63	-2.7 (5.8) n=56	-0.03	-0.21 to 0.14	0.715
BOSCC – RRB	0.4 (3.1) n=63	-0.2 (3.2) n=56	-0.08	-0.25 to 0.10	0.368
ADOS-2 – CSS	0.0 (1.8) n=61	-0.1 (1.7) n=54	0.05	-0.12 to 0.23	0.541
ADOS-2 – SA	-0.3 (2.1) n=61	-0.3 (1.9) n=54	0.09	-0.09 to 0.27	0.328
ADOS-2 -RRB	0.4 (1.7) n=61	0.3 (1.3) n=54	-0.01	-0.19 to 0.17	0.934
SRS16 parent	-2.7 (8.5) n=57	-0.2 (8.4) n=50	0.14	-0.03 to 0.30	0.080
RBS-R parent total score	-4.6 (15.0) n=55	0.9 (15.9) n=49	0.16	-0.01 to 0.33	**0.043**
RBS-R parent IS	-1.8 (8.6) n=56	2.0 (8.0) n=48	0.19	-0.01 to 0.36	**0.016**
RBS-R parent SI	0.1 (2.3) n=59	-0.2 (3.8) n=50	-0.05	-0.19 to 0.12	0.490
RBS-R parent SB	-1.6 (4.5) n=55	-1.0 (4.4) n=50	0.05	-0.10 to 0.22	0.488
RBS-R parent CB	-0.7 (4.1) n=57	0.6 (4.7) n=49	0.17	0.0 to 0.34	**0.025**
Developmental age [months]	7.6 (7.7) n=52	6.0 (6.2) n=46	0.09	-0.11 to 0.28	0.378
Language develop-ment [months]	8.6 (8.8) n=60	6.2 (8.6) n=50	0.01	-0.20 to 0.21	0.942
Full-scale IQ/DQ	2.1 (10.3) n=52	-2.3 (8.6) n=46	0.09	-0.11 to 0.28	0.422
Non-verbal IQ/DQ	-0.0 (12.8) n=60	-1.1 (13.7) n=52	-0.09	-0.29 to 0.10	0.386
Verbal IQ/DQ	3.2 (11.5) n=52	-1.1 (10.2) n=47	0.06	-0.13 to 0.26	0.549
CBCL ER	-1.3 (3.2) n=59	-0.4 (3.4) n=48	0.13	-0.04 to 0.31	0.100
CBCL AD	-0.3 (2.5) n=59	-0.2 (3.2) n=50	-0.01	-0.17 to 0.18	0.892
CBCL SP	-0.4 (2.2) n=59	-0.6 (2.9) n=48	-0.05	-0.23 to 0.13	0.482
CBCL SW	-1.5 (2.9) n=59	-0.6 (3.3) n=49	0.09	-0.08 to 0.26	0.279
CBCL AP	-0.9 (1.5) n=59	-0.1 (1.9) n=49	0.10	-0.07 to 0.28	0.195
CBCL AB	-2.4 (6.0) n=59	-0.5 (6.2) n=50	0.15	-0.01 to 0.33	0.064
CBCL SLEEP	-0.4 (2.3) n=59	-1.0 (3.0) n=50	-0.09	-0.27 to 0.07	0.252
BRIEF-P parent IS	-0.1 (8.8) n=57	2.3 (9.0) n=47	0.15	-0.04 to 0.34	0.087
BRIEF-P parent FL	-0.3 (7.3) n=57	1.2 (7.8) n=48	0.11	-0.08 to 0.29	0.228
BRIEF-P parent EM	-1.8 (8.2) n=54	2.9 (8.2) n=46	0.25	-0.05 to 0.44	**0.008**

Legend supplementary table 7

#The effect size's confidence interval was obtained via bootstrap and may hence not be fully coherent with the p-value.

Abbreviations:

PAS: primary analysis set, i.e. children with BOSCC measure at T2 (start), and at least one BOSCC measure at T4 (6 months) or T6 (12 months)

A-FFIP=intervention group; EIAU=early intervention as usual.

ADOS-2: Autism Diagnostic Observation Schedule – version 2, (sub-)scales CSS: calibrated severity score, SA: social affect, RRB: repetitive behaviour;

BOSCC: Brief Observation of Social Communication Change, subscales SC: social communication, RRB: repetitive behaviour;

BRIEF-P: Behaviour Rating Inventory of Executive Function - Preschool Version, subscales IS: inhibitory self-control, FL: flexibility, EM: emerging meta-cognition;

CBCL: Child Behaviour Checklist, subscales ER: emotional reactivity, AD: anxious-depressed, SP: somatic problems, SW: social withdrawal, AP: attention problems, AB: aggressive behaviour, SLEEP: sleeping problems;

DQ: developmental quotient, IQ: intelligence quotient;

RSB-R: Repetitive Behaviour Scale-revised, subscales IS: insistence on sameness, SI: self-injury, SB: stereotyped behaviour, CB: compulsive behaviour;

SRS16: Social Responsiveness Scale, 16 item version;

n: number, SD: standard deviation.

Supplementary table 8: Primary analysis of intervention effects x time in days

Effect			Effect estimate	Std. error	DF	t statistic	P value
Intercept	_	_	-1.2132	3.7543	143	-0.32	0.7471
Treatment	A-FFIP	_	-0.9082	1.9243	205	-0.47	0.6374
Treatment	EIAU	_	0	.	.	.	.
Time [weeks]	_	_	-0.04545	0.02619	135	-1.74	0.0850
Interaction: treatment*time	A-FFIP	_	0.03195	0.03654	131	0.87	0.3835
Interaction: treatment*time	EIAU	_	0	.	.	.	.
BOSCC-Total at T2	_	_	0.9064	0.05260	115	17.23	<.0001
Age (BOSCC T2)	_	_	0.05521	0.05211	118	1.06	0.2915
Center	_	Augsburg	-0.4657	1.6843	117	-0.28	0.7827
Center	_	Dresden	1.8631	2.0060	117	0.93	0.3549
Center	_	Frankfurt	1.9877	1.4256	117	1.39	0.1658
Center	_	Würzburg	0	.	.	.	.

Legend supplementary table 8:
A-FFIP=intervention group; EIAU=early intervention as usual,
BOSCC: Brief Observation of Social Communication Change.

Supplementary table 9: Sensitivity analysis with discrete measurement times

Effect			Effect estimate	Std. error	DF	t statistic	P value
Intercept	_	_	-3.9590	3.5775	121	-1.11	0.2706
Treatment	A-FFIP	_	1.0698	1.1996	118	0.89	0.3743
Treatment	EIAU	_	0	.	.	.	.
Visit T4	_	_	1.5523	0.7908	120	1.96	0.0520
Visit T6	_	_	0	.	.	.	.
Interaction: treatment*visit T4	A-FFIP	_	-0.9816	1.0888	119	-0.90	0.3691
Interaction: treatment*visit T6	A-FFIP	_	0	.	.	.	.
Interaction: treatment*visit T4	EIAU	_	0	.	.	.	.
Interaction: treatment*visit T6	EIAU	_	0	.	.	.	.
BOSCC-Total at T2	_	_	0.9064	0.05297	117	17.11	<.0001
Age (BOSCC T2)	_	_	0.05221	0.05210	117	1.00	0.3184
Center	_	Augsburg	-0.4877	1.6919	118	-0.29	0.7737
Center	_	Dresden	1.8642	2.0141	117	0.93	0.3566
Center	_	Frankfurt	1.9812	1.4328	118	1.38	0.1694
Center	_	Würzburg	0	.	.	.	.

Legend supplementary table 9:
A-FFIP=intervention group; EIAU=early intervention as usual,
BOSCC: Brief Observation of Social Communication Change.

Supplementary table 10: Sensitivity analysis - primary analysis model adjusted for treatment interruption effect

Effect				Effect estimate	Std. error	DF	t statistic	P value
Intercept	_	_	_	-2.5160	3.7041	140	-0.68	0.4981
Treatment	A-FFIP	_	_	-0.9766	1.9103	203	-0.51	0.6097
Treatment	EIAU	_	_	0	.	.	.	.
Time [weeks]	_	_	_	-0.05315	0.02633	132	-2.02	**0.0455**
Interaction: treatment*time	A-FFIP	_	_	0.03504	0.03652	132	0.96	0.3390
Interaction: treatment*time	EIAU	_	_	0	.	.	.	.
BOSCC-Total at T2	_	_	_	0.9210	0.05160	115	17.85	<.0001
Age (BOSCC T2)	_	_	_	0.04548	0.05097	117	0.89	0.3740
Center	_	Augsburg	_	0.5010	1.6839	117	0.30	0.7666
Center	_	Dresden	_	2.0010	1.9575	116	1.02	0.3088
Center	_	Frankfurt	_	2.5313	1.4060	116	1.80	0.0744
Center	_	Würzburg	_	0	.	.	.	.
Treatment interruption	_	_	Yes	2.7132	1.0317	121	2.63	**0.0097**
Treatment interruption	_	_	No	0	.	.	.	.

Legend supplementary table 10:
A-FFIP=intervention group; EIAU=early intervention as usual,
BOSCC: Brief Observation of Social Communication Change.

Supplementary table 11: Sensitivity analysis - primary analysis model adjusted for number of A-FFIP/EIAU intervention sessions

Effect			Effect estimate	Std. error	DF	t statistic	P value
Intercept	_	_	-1.0992	3.8462	140	-0.29	0.7755
Treatment	A-FFIP	_	3.0672	4.5104	150	0.68	0.4975
Treatment	EIAU	_	0	.	.	.	.
Time [weeks]	_	_	-0.04607	0.02620	134	-1.76	0.0810
Interaction: treatment*time	A-FFIP	_	0.03188	0.03655	131	0.87	0.3847
Interaction: treatment*time	EIAU	_	0	.	.	.	.
BOSCC-Total at T2	_	_	0.9022	0.05285	114	17.07	<.0001
Age (BOSCC T2)	_	_	0.05465	0.05316	116	1.03	0.3061
Number of sessions	_	_	0.01886	0.02295	116	0.82	0.4128
Interaction: treat.*sessions	A-FFIP	_	-0.06784	0.06017	117	-1.13	0.2619
Interaction: treat.*sessions	EIAU	_	0	.	.	.	.
Center	_	Augsburg	-0.6521	1.7058	115	-0.38	0.7030
Center	_	Dresden	1.5500	2.0280	115	0.76	0.4463
Center	_	Frankfurt	1.5433	1.4766	116	1.05	0.2981
Center	_	Würzburg	0	.	.	.	.

Legend supplementary table 11:
A-FFIP=intervention group; EIAU=early intervention as usual,
BOSCC: Brief Observation of Social Communication Change.

Supplementary table 12: Sensitivity analysis - primary analysis model on the per protocol set

Effect			Effect estimate	Std. error	DF	t statistic	P value
Intercept	_	_	0.8964	4.7024	85.4	0.19	0.8493
Treatment	A-FFIP	_	1.0159	2.4985	145	0.41	0.6849
Treatment	EIAU	_	0	.	.	.	.
Time [weeks]	_	_	-0.04186	0.02579	85.5	-1.62	0.1082
Interaction: treatment*time	A-FFIP	_	-0.03300	0.04509	85.1	-0.73	0.4663
Interaction: treatment*time	EIAU	_	0	.	.	.	.
BOSCC-Total at T2	_	_	0.9370	0.06411	72.6	14.62	<.0001
Age (BOSCC T2)	_	_	0.002480	0.06681	74.2	0.04	0.9705
Center	_	Augsburg	0.02859	1.9035	73.3	0.02	0.9881
Center	_	Dresden	0.8613	2.3367	73.3	0.37	0.7135
Center	_	Frankfurt	1.2200	1.8879	73.2	0.65	0.5201
Center	_	Würzburg	0	.	.	.	.

Legend supplementary table 12:
A-FFIP=intervention group; EIAU=early intervention as usual,
BOSCC: Brief Observation of Social Communication Change.

Supplementary table 13: Sensitivity analysis - primary analysis model on the FAS with complete data

Effect			Effect estimate	Std. error	DF	t statistic	P value
Intercept	_	_	-1.1662	3.8305	138	-0.30	0.7612
Treatment	A-FFIP	_	-1.1343	1.9695	203	-0.58	0.5653
Treatment	EIAU	_	0	.	.	.	.
Time [weeks]	_	_	-0.04741	0.02656	129	-1.78	0.0766
Interaction: treatment*time	A-FFIP	_	0.03436	0.03691	127	0.93	0.3536
Interaction: treatment*time	EIAU	_	0	.	.	.	.
BOSCC-Total at T2	_	_	0.9070	0.05359	111	16.92	<.0001
Age (BOSCC T2)	_	_	0.05889	0.05312	113	1.11	0.2699
Center	_	Augsburg	-0.6384	1.7312	111	-0.37	0.7130
Center	_	Dresden	1.7377	2.0468	112	0.85	0.3977
Center	_	Frankfurt	1.9397	1.4755	111	1.31	0.1914
Center	_	Würzburg	0	.	.	.	.

Legend supplementary table 13:
A-FFIP=intervention group; EIAU=early intervention as usual,
BOSCC: Brief Observation of Social Communication Change.

Supplementary table 14: Teacher-rated secondary outcomes – effect sizes

**Outcome**	**A-FFIP (PAS N=64) mean change T1/T2 to T6 (SD)**	**EIAU (PAS N=60) mean change T1/T2 to T6 (SD)**	**Adjusted effect size**	**95%-confidence interval #**	**p-value**
SRS16 teacher	-3.7 (7.7) n=42	-0.9 (8.5) n=30	0.10	-0.11 to 0.33	0.350
RBS-R teacher total score	-4.3 (22.0) n=35	-2.6 (15.3) n=28	0.01	-0.23 to 0.26	0.945
RBS-R teacher IS	0.7 (10.2) n=19	-2.4 (7.7) n=20	-0.17	-0.46 to 0.13	0.280
RBS-R teacher SI	-0.3 (3.3) n=45	-0.3 (3.1) n=33	0.04	-0.18 to 0.33	0.689
RBS-R teacher SB	-2.1 (6.5) n=42	-0.8 (5.3) n=33	0.03	-0.18 to 0.25	0.753
RBS-R teacher CB	-0.6 (5.0) n=44	0.7 (3.6) n=33	0.14	-0.07 to 0.36	0.179
CTRF ER	-1.3 (3.2) n=43	-0.6 (2.9) n=29	0.14	-0.08 to 0.39	0.159
CTRF AD	-0.6 (3.4) n=41	0.1 (3.9) n=31	0.08	-0.13 to 0.32	0.431
CTRF SP	-0.4 (1.5) n=39	0.3 (1.5) n=31	0.27	-0.07 to 0.50	**0.011**
CTRF SW	-2.6 (4.2) n=43	-1.7 (3.9) n=31	0.09	-0.12 to 0.29	0.421
CTRF AP	-1.2 (3.6) n=43	-1.6 (3.6) n=31	-0.12	-0.33 to 0.11	0.266
CTRF AB	-0.7 (7.2) n=41	0.6 (7.9) n=30	0.14	-0.10 to 0.38	0.189
BRIEF-P teacher IS	-2.3 (10.3) n=44	0.8 (11.5) n=31	0.12	-0.10 to 0.34	0.270
BRIEF-P teacher FL	-3.3 (10.0) n=44	0.1 (8.7) n=29	0.21	-0.01 to 0.45	**0.045**
BRIEF-P teacher EM	-3.5 (11.0) n=37	-2.1 (13.0) n=27	-0.10	-0.34 to 0.14	0.410

Legend supplementary table 14

#The effect size’s confidence interval was obtained via bootstrap and may hence not be fully coherent with the p-value.

<u>Abbreviations:</u>

PAS: primary analysis set, i.e. children with BOSCC measure at T2 (start), and at least one BOSCC measure at T4 (6 months) or T6 /12 months)

A-FFIP=intervention group; EIAU=early intervention as usual.

BRIEF-P: Behaviour Rating Inventory of Executive Function - Preschool Version, subscales IS: inhibitory self-control, FL: flexibility, EM: emerging meta-cognition;

CTRF: Kindergarten Children Teacher Report Form, subscales ER: emotional reactivity, AD: anxious-depressed, SP: somatic problems, SW: social withdrawal, AP: attention problems, AB: aggressive behaviour;

RSB-R: Repetitive Behaviour Scale-revised, subscales IS: insistence on sameness, SI: self injury, SB: stereotyped behaviour, CB: compulsive behaviour;

SRS16: Social Responsiveness Scale, 16 item version;

n: number, SD: standard deviation.

Supplementary table 15: Teacher rated secondary outcomes in the full analysis set: Unadjusted T2, T4 and T6 measures

Outcome	A-FFIP T1/2 (mean, SD, missing)	EIAU T2 (mean, SD, missing)	A-FFIP T4 (mean, SD, missing)	EIAU T4 (mean, SD, missing)	A-FFIP T6 (mean, SD, missing)	EIAU T6 (mean, SD, missing)
SRS16 teacher	31.2 (9.5) 18	31.2 (10.0) 27	28.6 (9.0) 18	31.6 (10.1) 23	27.9 (9.5) 17	29.7 (10.4) 22
RBS-R teacher total score	30.9 (20.6) 24	29.3 (19.7) 30	25.6 (14.8) 19	32.8 (20.1) 27	25.8 (17.3) 20	30.3 (20.1) 24
RBS-R teacher IS	15.4 (10.4) 40	16.6 (10.8) 41	11.9 (8.5) 35	17.5 (11.1) 35	13.1 (9.4) 32	14.5 (10.2) 33
RBS-R teacher SI	1.8 (2.3) 15	2.0 (3.2) 23	1.1 (1.8) 16	2.7 (4.2) 22	1.5 (3.2) 16	1.7 (2.9) 21
RBS-R teacher SB	8.8 (6.1) 16	9.3 (6.1) 23	8.5 (5.7) 17	9.4 (6.1) 23	7.5 (5.5) 19	9.4 (5.9) 21
RBS-R teacher CB	4.1 (4.9) 16	3.8 (3.9) 25	3.6 (3.2) 16	4.9 (4.3) 22	3.4 (4.0) 17	4.2 (4.0) 21
CTRF ER	4.4 (3.0) 18	4.0 (2.6) 26	3.0 (2.1) 17	4.6 (3.0) 22	3.4 (2.6) 16	3.6 (2.4) 22
CTRF AD	3.7 (3.3) 20	2.9 (2.8) 25	2.7 (2.2) 16	3.6 (2.7) 23	3.0 (2.7) 17	3.2 (2.0) 22
CTRF SP	1.0 (1.2) 20	1.0 (1.0) 25	0.9 (1.2) 18	1.5 (1.6) 22	0.6 (0.9) 18	1.1 (1.5) 22
CTRF SW	10.8 (4.4) 19	10.4 (4.2) 25	8.9 (4.4) 17	9.9 (4.7) 24	8.0 (4.3) 16	8.6 (4.3) 25
CTRF AP	10.6 (4.0) 16	11.3 (3.7) 25	9.2 (4.4) 17	10.2 (4.3) 22	9.2 (5.0) 17	9.1 (4.6) 21
CTRF AB	11.8 (5.4) 20	12.1 (7.4) 26	10.4 (5.2) 18	13.9. (7.4) 24	10.8 (6.4) 17	12.5 (6.0) 22
BRIEF-P teacher IS	27.8 (11.0) 18	28.7 (12.7) 26	25.4 (10.1) 18	31.1 (11.8) 22	26.7 (11.5) 17	29.7 (11.2) 23
BRIEF-P teacher FL	19.7 (9.2) 17	21.1 (9.4) 27	17.1 (8.1) 19	22.0 (9.0) 23	17.8 (8.5) 16	20.0 (8.8) 23
BRIEF-P teacher EM	28.1 (12.8) 24	28.2 (12.9) 32	26.3 (12.2) 24	26.3 (14.3) 31	24.3 (13.3) 21	26.8 (13.1) 29

Legend supplementary table 15
The data shown in this table refer to the full analysis set of A-FFIP (n=68) and EIAU (n=66).
<u>Abbreviations:</u>
A-FFIP=intervention group; EIAU=early intervention as usual.
BRIEF-P: Behaviour Rating Inventory of Executive Function - Preschool Version, subscales IS: inhibitory self-control, FL: flexibility, EM: emerging meta-cognition;
CTRF: Kindergarten Children Teacher Report Form, subscales ER: emotional reactivity, AD: anxious-depressed, SP: somatic problems, SW: social withdrawal, AP: attention problems, AB: aggressive behaviour;
RSB-R: Repetitive Behaviour Scale-revised, subscales IS: insistence on sameness, SI: self injury, SB: stereotyped behaviour, CB: compulsive behaviour;
SRS16: Social Responsiveness Scale, 16 item version;
n: number, SD: standard deviation.

Supplementary table 16: Parent- and family-related secondary outcomes – effect sizes

Outcome	A-FFIP (PAS N=64) mean change T1/T2 to T6 (SD)	EIAU (PAS N=60) mean change T1/T2 to T6 (SD)	Adjusted effect size	95%-confidence interval #	p-value
PSOC mother	1.4 (3.4) n=57	0.6 (3.5) n=46	0.05	-0.14 to 0.23	p=0.578
PSOC father	1.4 (4.5) n=46	-0.2 (4.5) n=41	0.08	-0.10 to 0.23	p=0.414
DASS-21 stress mother	-1.7 (3.9) n=57	-0.7 (4.1) n=47	0.14	-0.04 to 0.33	p=0.105
DASS-21 depression mother	-0.8 (3.4) n=57	0.0 (4.9) n=45	0.13	-0.06 to 0.31	p=0.146
DASS-21 anxiety mother	-0.4 (3.8) n=57	-0.1 (3.2) n=46	0.03	-0.14 to 0.25	p=0.720
DASS-21 stress father	-0.4 (3.9) n=47	-0.7 (5.0) n=40	-0.06	-0.23 to 0.12	p=0.500
DASS-21 depression father	-0.7 (3.7) n=47	0.1 (4.7) n=40	0.03	-0.14 to 0.21	p=0.736
DASS-21 anxiety father	0.4 (4.5) n=47	0.3 (4.5) n=40	-0.01	-0.21 to 0.18	p=0.927
FQOLS	-1.6 (11.1) n=55	4.0 (9.8) n=50	-0.14	-0.31 to 0.04	p=0.170

Legend supplementary table 16:
#The effect size's confidence interval was obtained via bootstrap and may hence not be fully coherent with the p-value.
Abbreviations:
DASS-21: Depression, Anxiety and Stress Scales – short form
DCMA: Dyadic Communication Measure for Autism – Revised
FQOLS: Family related quality of life
PSOC: Parenting Sense of Competence
n: number, SD: standard deviation

Supplementary table 17: Parent- and family-related secondary outcomes in the full analysis set: Unadjusted T2, T4 and T6 measures

Outcome	A-FFIP T1/2 (mean, SD, missing)	EIAU T2 (mean, SD, missing)	A-FFIP T4 (mean, SD, missing)	EIAU T4 (mean, SD, missing)	A-FFIP T6 (mean, SD, missing)	EIAU T6 (mean, SD, missing)
PSOC mother	18.2 (4.6) 2	19.2 (4.0) 13	18.4 (4.6) 7	19.2 (4.2) 14	19.4 (4.2) 8	19.4 (4.3) 29
PSOC father	17.0 (4.7) 9	18.5 (4.2) 18	17.3 (4.6) 13	17.3 (5.0) 20	18.0 (4.3) 17	19.0 (5.0) 20
DASS-21 stress mother	7.3 (5.1) 2	7.1 (4.4) 13	5.7 (4.9) 9	6.7 (5.0) 17	5.5 (4.7) 9	6.3 (4.9) 14
DASS-21 depression mother	4.2 (4.0) 2	4.1 (4.5) 15	3.4 (3.9) 9	4.1 (4.4) 17	3.3 (4.2) 9	4.0 (5.2) 14
DASS-21 anxiety mother	2.2 (3.1) 2	2.7 (4.0) 14	2.0 (3.6) 9	2.1 (2.7) 17	1.9 (3.7) 9	2.7 (3.4) 14
DASS-21 stress father	6.0 (4.8) 7	5.5 (4.3) 18	5.3 (5.2) 12	5.0 (4.2) 21	5.7 (5.5) 17	4.7 (4.5) 20
DASS-21 depression father	3.8 (4.6) 7	2.5 (3.3) 18	3.7 (5.3) 13	2.4 (3.0) 21	3.6 (5.0) 17	2.6 (4.3) 20
DASS-21 anxiety father	2.1 (3.6) 7	1.8 (3.5) 18	2.1 (4.4) 13	1.8 (3.8) 21	2.6 (4.7) 17	2.1 (4.6) 20
FQOLS	52.4 (10.3) 1	48.7 (9.4) 1			50.7 (10.5) 12	52.4 (12.0) 16

Legend supplementary table 17:
The data shown in this table refer to the full analysis set of A-FFIP (n=68) and EIAU (n=66).
Abbreviations:
DASS-21: Depression, Anxiety and Stress Scales – short form
DCMA: Dyadic Communication Measure for Autism – Revised
FQOLS: Family related quality of life
PSOC: Parenting Sense of Competence
n: number, SD: standard deviation

Table 18: Moderation analysis: age at T2

Effect			Effect estimate	Std. error	DF	t statistic	P value
Intercept	_	_	15.1950	7.8512	221	1.94	0.0542
Treatment	A-FFIP	_	-22.1885	9.8641	203	-2.25	0.0256
Treatment	EIAU	_	0	.	.	.	.
Time [weeks]	_	_	-0.3139	0.1379	129	-2.28	0.0245
Interaction: treatment*time	A-FFIP	_	0.3497	0.1863	128	1.88	0.0628
Interaction: treatment*time	EIAU	_	0	.	.	.	.
Age (BOSCC T2)	_	_	-0.2667	0.1455	208	-1.83	0.0683
Interaction: treat.*age	A-FFIP	_	0.4240	0.1927	203	2.20	0.0289
Interaction: treat.*age	EIAU	_	0	.	.	.	.
Interaction: time*age	_	_	0.005333	0.002696	130	1.98	0.0500
Interaction: treat.*time*age	A-FFIP	_	-0.00633	0.003624	128	-1.75	0.0832
Interaction: treat.*time*age	EIAU	_	0	.	.	.	.
BOSCC-Total at T2	_	_	0.9025	0.05227	114	17.26	<.0001
Center	_	Augsburg	-0.4010	1.6740	116	-0.24	0.8111
Center	_	Dresden	1.8372	1.9911	115	0.92	0.3581
Center	_	Frankfurt	1.8004	1.4227	117	1.27	0.2082
Center	_	Würzburg	0	.	.	.	.

Legend supplementary table 18:
A-FFIP=intervention group; EIAU=early intervention as usual,
BOSCC: Brief Observation of Social Communication Change.
