# Supplementary material for: Complex, low‐intensity, individualised naturalistic developmental behavioural intervention in toddlers and pre‐schoolers with autism spectrum disorder: The multicentre, observer‐blind, parallel‐group randomised‐controlled A‐FFIP trial
Source: J Child Psychol Psychiatry. 2025 Mar 26;66(10):1500–13. doi: 10.1111/jcpp.14162 (PMC12447682; doi:10.1111/jcpp.14162)
Supplement: Supplementary file 3 — Appendix S3. Full list of staff. [file JCPP-66-1500-s001.pdf]

Complex, low intensity, individualised naturalistic developmental behavioural intervention in tod¬dlers and preschoolers with Autism Spectrum Disorder: The multicentre, observer-blind, parallel-group randomised controlled A-FFIP trial
Christine M. Freitag (1), Marietta Kirchner (2), Lukas D. Sauer (2), Solveig K. Kleber (1), Leonie Polzer (1), Naisan Raji (1), Christian Lemler (1), Ulrike Fröhlich (3), Julia Geissler (4), Melanie Ring (5), Veit Roessner (5), Regina Taurines (4), Michelle Noterdaeme (3), Karoline Teufel (1), Ziyon Kim (1)*, Janina Kitzerow-Cleven(1)*
*** joint last authors**

Full list of staff supporting the trial

Coordinating investigator and trial management	Prof. Dr. Christine M. Freitag
Local coordinating investigator	Prof. Dr. Christine M. Freitag (Frankfurt)
	Prof. Dr. Michele Noterdaeme (until 2022) &
	Dr. Tomasz Jarczok (since 2023) (Augsburg)
	Prof. Dr. Marcel Romanos (Würzburg)
	Prof. Dr. Veit Rößner (Dresden)
Overall study management	Prof. Dr. Christine M. Freitag, Dr. Janina Kitzerow-Cleven &
	Dr. Ziyon Kim
Local study management (Head)	Dr. Ziyon Kim & Dr. Janina Kitzerow- Cleven (Frankfurt)
	Ulrike Fröhlich (Augsburg)
	Dr. Regina Taurines (Würzburg)
	Dr. Katja Albertowski (Dresden)
Data Safety Monitoring Board (DSMB)	Susanne Lezius, MSc
	Prof. Dr. Christina Stadler
	Prof. Dr. Hans-Christoph Steinhausen
Trial statistican	Institut für Medizinische Biometrie und Statistik (IMBI):
	Prof. Dr. Meinhard Kieser (Supervising Biometrician)
	Dr. Marietta Kirchner (Responsible Biometrician)
	Lukas Sauer (Representative of the responsible Biometrician)
	Jolanda Brezinski (Datamanagement)
Monitoring	Dr. Anuradha Sharma (Coordination Center for Clinical Trials)
	Dr. Janina Kitzerow-Cleven (Pre-Trial Visits)

List of each participating study cites

**Frankfurt**	Local coordinating project management	Diagnostic, Assessment across measurement timepoints & Video ratings	A-FFIP Therapy (Main Therapist)	A-FFIP Therapy (Co-Therapist)	A-FFIP Supervison & Manual Adherence/Therapist Fidelity ratings	Student assisstant/Research assisstant
	Solvejg Kleber	Solvejg Kleber	Laura Rubio	Kathrin Hörnlen	Karoline Teufel	Luisa Schnettler
	Leonie Polzer	Leonie Polzer	Jessica Betsch	Julia Nakissa	Eva Lopez	Manuela Martic
	Christian Lemler	Christian Lemler	Susanne Hansen	Lea Benzing	Monika Schneider	Ranfei Wang
	Naisan Raji	Naisan Raji	Leyla Elsuni	Julia Haller	Leyla Elsuni	Maren Schimpke
	Luisa Schnettler	Dr. Janina Kitzerow-Cleven	Dr. Michael Sachse	Emilia Schramm	Dr. Janina Kitzerow-Cleven	Anna Kurzke
			Monika Schneider	Manuel Jung	Dr. Michael Sachse	Sophia Müller
			Rebecca Tous	Felix Schawe	Laura Rubio	Marvin Dunkel
			Dr. Ziyon Kim	Maike Schatz	Dr. Ziyon Kim	Viktoria Kohl
			Rieke Klünker	Stefan Göbel		Samuel Mais
			Veronika Banknowksi	Robin Kirchner		Irina Kegler
			Sohpie Soll	Sarah Theis		Charlotte Schneider
			Eva Lopez	Andrea Schmidt		Philipp Hengst
				Christin Schwarz		Margerita Slivko
				Felix Hensel		Anton Daser
				Selin Yazici		Kathalin Lechner
				Lorenz Lowis		
				Laurena Wagner		
				Sabine Becker		
				Lorena Bröring		
				Henrike Stroh		

**Augsburg**	Local coordinating project management	Diagnostic & Assessment across measurement timepoints	A-FFIP Therapy (Main Therapist)	A-FFIP Therapy (Co-Therapist)		Student assisstant/Research assisstant
	Maria Bauer	Stefanie Rompe	Paula Grüner	Michael Kempter		Maria Schell
	Stefanie Besold	Jana Maier	Anna Gumpel	Maria Schell		Silke Tobiasz
		Theresa Kral	Natalie Oberlies	Silke Tobiasz		Dr. Sarah Lippe
		Marisa Back	Malin Schulze	Dr. Sarah Lippe		Ronja Koch
			Antje Brandhorst	Ronja Koch		
			Ulrike Fröhlich			

**Würzburg**	Local coordinating project management	Diagnostic & Assessment across measurement timepoints	A-FFIP Therapy (Main Therapist)	A-FFIP Therapy (Co-Therapist)		Student assisstant/Research assisstant
	Anke Leben	Anke Leben	Anke Leben	Maria Mann		Diana Hauer
	Lisa Haber	Lisa Haber	Patricia Dengler	Patricia Döll		Lea Zwilling
	Elke Hack	Anika Rüger	Dr. Julia Geissler	Laura Mazurowski		Lea Brosig
	Dr. Julia Geissler	Anna Harzdorf	Antonia Stanean	Ulrich Neuner		Maria Mann
	Alena Burlein	Christin Heim	Lisa Haber	Philipp Schüssler		Laura Mazurowski
	Annette Nowak	Patricia Dengler		Valerie Jaklin		Ulrich Neuner
		Nina Müller-Keil		Julia Jaksch		Philipp Schüssler
		Sissi Falgner				Valerie Jaklin
		Antonia Stanean				Julia Jaksch
		Marie-Pierre Minten				Friederike Feithen
		Louise Haslam				Lisa Gierer
		Bernd Werner				

**Dresden**	Local coordinating project management	Diagnostic & Assessment across measurement timepoints	A-FFIP Therapy (Main Therapist)	A-FFIP Therapy (Co-Therapist)		Student assisstant/Research assisstant
	Christina Kappler-Friedrichs	Judith Mack	Dr. Melanie Ring	Ann-Christin Schröder		Franka Foth
	Petra Boettge	Sophie Schächer	Susanne Hollborn-Stewart	Andrea Federolf		Lilly Allner
	Dr. Melanie Ring	Marieluise Herrmann	Lena Zimmermann			Jasmin Gora
	Dr. Nicole Wolff	Teresa Heilig	Sina Cruse			Leonie Klug
						Josefin Mesow
						Susann Meltzer
						Pauline Arnold
